# Regulation of a progenitor gene program by SOX4 is essential for mammary tumor proliferation

**DOI:** 10.1038/s41388-021-02004-z

**Published:** 2021-09-28

**Authors:** M. Guy Roukens, Cynthia L. Frederiks, Danielle Seinstra, Luca Braccioli, Antoine A. Khalil, Cornelieke Pals, Simon De Neck, Laura Bornes, Evelyne Beerling, Michal Mokry, Alain de Bruin, Bart Westendorp, Jacco van Rheenen, Paul J. Coffer

**Affiliations:** 1 Regenerative Medicine Center Utrecht, University Medical Center Utrecht, Utrecht, The Netherlands; 2 Center for Molecular Medicine Utrecht, University Medical Center Utrecht, Utrecht, The Netherlands; 3 Department of Molecular Pathology, Oncode Institute, the Netherlands Cancer Institute, Amsterdam, The Netherlands; 4 Department Biomolecular Health Sciences, Faculty of Veterinary Medicine, Utrecht University, Utrecht, The Netherlands; 5 Department of Pediatrics, University Medical Center Groningen, University of Groningen, Groningen, The Netherlands; 6 Laboratory of Experimental Cardiology, University Medical Center Utrecht, Utrecht, The Netherlands

**Keywords:** Breast cancer, Cancer stem cells

## Abstract

In breast cancer the transcription factor SOX4 has been shown to be associated with poor survival, increased tumor size and metastasis formation. This has mostly been attributed to the ability of SOX4 to regulate Epithelial-to-Mesenchymal-Transition (EMT). However, SOX4 regulates target gene transcription in a context-dependent manner that is determined by the cellular and epigenetic state. In this study we have investigated the loss of SOX4 in mammary tumor development utilizing organoids derived from a PyMT genetic mouse model of breast cancer. Using CRISPR/Cas9 to abrogate SOX4 expression, we found that SOX4 is required for inhibiting differentiation by regulating a subset of genes that are highly activated in fetal mammary stem cells (fMaSC). In this way, SOX4 re-activates an oncogenic transcriptional program that is regulated in many progenitor cell-types during embryonic development. SOX4-knockout organoids are characterized by the presence of more differentiated cells that exhibit luminal or basal gene expression patterns, but lower expression of cell cycle genes. In agreement, primary tumor growth and metastatic outgrowth in the lungs are impaired in SOX4^KO^ tumors. Finally, SOX4^KO^ tumors show a severe loss in competitive capacity to grow out compared to SOX4-proficient cells in primary tumors. Our study identifies a novel role for SOX4 in maintaining mammary tumors in an undifferentiated and proliferative state. Therapeutic manipulation of SOX4 function could provide a novel strategy for cancer differentiation therapy, which would promote differentiation and inhibit cycling of tumor cells.

## Introduction

An underlying aspect of cellular plasticity in tumorigenesis is the re-activation of developmental pathways by tumor cells [1, 2], of which perhaps the best characterized is epithelial-to-mesenchymal-transition (EMT). During EMT epithelial cells lose their tight junctions and gain migratory and invasive properties [3, 4]. EMT has been suggested to be of major importance for the metastatic cascade by facilitating detachment from the primary tumors and invasion into the surrounding stroma. However, as most secondary tumors exhibit epithelial characteristics this suggested that tumor cells require to undergo the reversal of EMT, mesenchymal-to-epithelial transition (MET) for efficient metastatic outgrowth. Indeed, reverting EMT in circulating tumor cells was shown to promote metastatic outgrowth in animal models [5–7]. We have shown, using a real-time EMT reporter in mammary tumors of a PyMT genetic mouse model that cells exist in a spectrum of EMT states. Moreover, metastatic outgrowths regain E-cadherin levels before they can grow out to macrometastases [8, 9]. These studies indicate that EMT is dynamically regulated during metastasis formation and that mesenchymal cells are unable to sustain tumor growth.

Cellular plasticity within tumors is further driven by a variety of differentiation states. These states resemble stem/progenitor cells and the differentiated cell types of the organ where the tumor originated from. Cells with enhanced stem- or progenitor cell abilities exhibit the capacity to fuel the growth of tumors, while terminally differentiated cells within tumors have poor potential to drive tumor growth [10–12]. For breast cancer, several groups have reported a correlation between expression profiles of mammary stem/progenitor cells and human breast cancers [13–15]. In particular, fetal mammary stem cells (fMaSC) exhibit marked similarities to aggressive human breast cancers [13, 14]. This suggests that mammary tumor development may be supported by re-activation of fMaSC gene expression programs. How these fMaSC-like genes contribute to the ability to fuel tumor growth is currently unknown.

The transcription factor SOX4 has been found to be one of the most frequently upregulated genes in a variety of solid and hematological cancers [16]. An increasing number of studies attest to an important role for SOX4 in breast cancer. SOX4 protein expression correlates with tumor size, mitotic index and poor prognosis of breast cancer patients [17]. Initial studies have suggested that SOX4 is involved in mediating invasion and migration [18–20]. We and others have shown that SOX4 mediates EMT in mammary epithelial cells [21–23]. In accordance, knockdown of SOX4 leads to impaired metastasis formation [17, 18, 23] and in some studies, reduced primary tumor growth [22, 23]. A caveat to most of these studies is that it has been shown that SOX4 regulates EMT in vitro and mammary tumor progression in vivo. As the relevance of EMT to tumor growth is unclear [4, 24] it raises the question whether SOX4 affects tumor growth independently of regulating EMT.

Here we have interrogated the role of SOX4 in breast cancer using organoids derived from a *MMTV-PyMT; MMTV-Cre; Ecadherin-mCFP* mouse model. Up to now the role of SOX4 in breast cancer has been studied in cell lines consisting of untransformed epithelial cells or in basal/mesenchymal-like tumor cells [17, 21–23]. The PyMT organoids model a distinct molecular subtype as they form luminal ductal mammary tumors upon orthotopic transplantation. Moreover, these organoids enable interrogation of EMT in vivo using the E-cadherin-mCFP reporter. Unexpectedly, we observed that deletion of SOX4 from PyMT tumors does not inhibit EMT. Instead, we found that SOX4 impairs differentiation and regulates fMaSC genes. Furthermore, SOX4 activates a cell cycle gene expression program that shares gene sets with many progenitor cell types and primes the cells for proliferation in vivo. Consequently, loss of SOX4 leads to a strong impairment of tumor growth in both the mammary fat pad and in the lungs.

Together, this study uncovers a novel mechanism by which SOX4 regulates a progenitor cell cycle program that is crucial for propagation in breast cancer. Therapeutic manipulation of SOX4 may thus provide a novel approach to interfere with tumor propagating cells.

## Results

### Loss of SOX4 in murine PyMT organoids inhibits primary tumor formation and metastasis formation

Tumor-organoids were derived from a *MMTV-PyMT; MMTV-Cre; Ecadherin-mCFP* murine breast cancer model. To explore how SOX4 affects mammary tumor progression in this model, we generated SOX4 knockout organoid lines (SOX4^KO^). We selected one control organoid line and two SOX4^KO^ organoid lines that were found to contain a single (bi-allelic) indel (Supplementary Fig. 1A). By western blotting we confirmed the absence of SOX4 protein (Supplementary Fig. 1B). In vitro the SOX4^KO^ lines exhibited a small increase in proliferation compared to control cells (Supplementary Fig. 1C). In addition, when plated as single cells SOX4^KO^ organoids showed a small growth advantage when growing out into (multicellular) organoids, although the average organoid size was unaffected (Supplementary Fig. 1D–E).

**Fig. 1:**
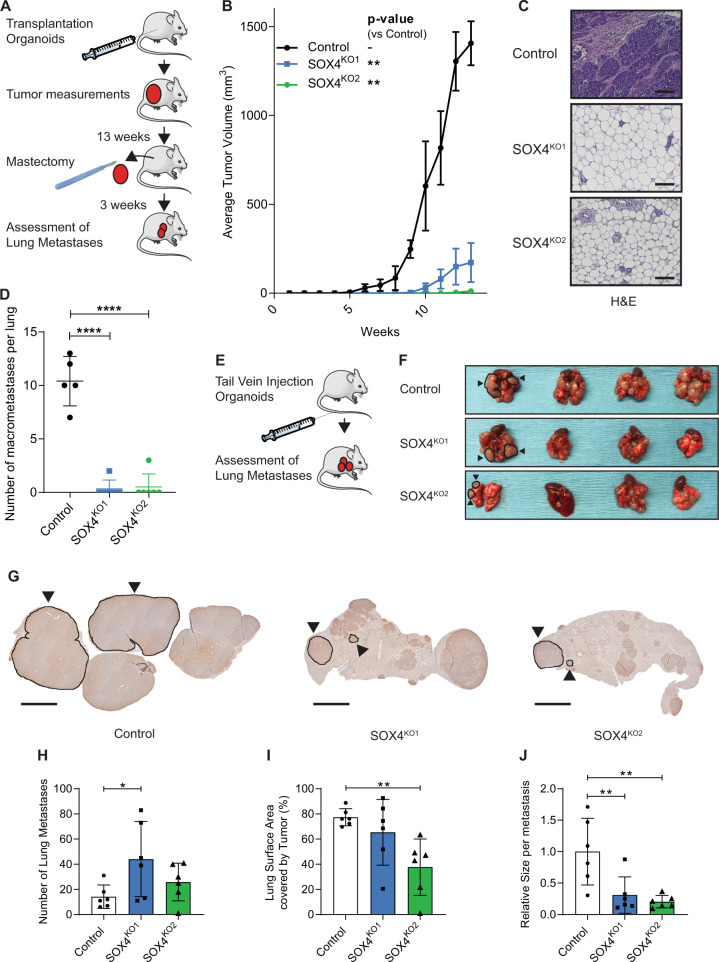
Loss of SOX4 in murine PyMT organoids inhibits primary tumor formation and metastasis formation. **A** Schematic representation of setup of primary tumor experiment. **B** Growth curves for control and SOX4^KO^ organoids after mammary transplantation. Data represented as mean volume (mm^3^). Error bars represent standard error of the mean (SEM). P-values were determined by the “compare growth curves” method [41]. **C** Immunohistochemical images showing H&E staining for isolated tumors/glands. Scale bar is 100 µm. **D** Number of macrometastases in lungs of mice as determined by eye by 2 scientists. **E** Schematic representation of experimental setup for tail vein experiment. Organoids were injected into the tail veins of recipient mice. Mice were sacrificed and lungs were isolated and assessed for metastases outgrowth. **F** Images showing lungs that were isolated after tail vein experiment. Examples of macrometastases are highlighted by black circles and arrowheads. **G** Representative images of CFP-staining on paraffin sections to identify metastases in lungs. Examples of metastatic lesions are highlighted by black encirclement and arrowheads. Scale bar = 1 mm. **H** Quantification of the number of lung macrometastases per field of view. **I** Quantification of surface area of lungs covered by tumors expressed in percentage. **J** Relative size per macrometastasis. Data in **D**–**J** is represented as average ± SD. *P*-values were calculated by ANOVA using Dunnett test for multiple comparisons (*p < 0.05, **p < 0.01, ****p-value < 0.0001).

Organoids were transplanted into the mammary fat pad of immunodeficient NSG mice and tumor growth was followed for 16 weeks (Fig. 1A). We observed that loss of SOX4 significantly impaired primary tumor growth (Fig. 1B). All mice that were transplanted with control organoids (5/5) developed mammary tumors. In contrast, the mice transplanted with SOX4^KO^ organoids exhibited a substantially lower tumor outgrowth (3/6 for SOX4 ^KO1^ and 1/6 for SOX4 ^KO2^; Supplementary Fig. 1F–G). In mice that didn’t exhibit palpable tumor growth we also could not identify tumors by immunohistochemistry (Fig. 1C). When tumors reached a size of 1000 mm^3^ a mastectomy was performed and animals were left for an additional 3 weeks to analyze metastatic outgrowth (Fig. 1A). None of the SOX4^KO^ tumors reached this size, but a mastectomy was performed on one of the SOX4^KO^ tumors albeit at a lower volume (576 mm^3^) (Supplementary Fig. 1F). These analyses showed the number of (macro)metastases in the lungs were very low or absent in mice transplanted with SOX4^KO^ organoids (Fig. 1D).

Since a majority of mice injected with SOX4^KO^ organoids did not have a primary tumor we performed an alternative experiment to explore whether SOX4 also directly affects metastatic outgrowth. Tumor organoids were injected into the tail vein, and metastases are therefore not influenced by the size of a primary tumor (Fig. 1E). We observed that loss of SOX4 reduces the size of metastatic lesions (Fig. 1F). We performed CFP immunostaining to more accurately quantify the metastases (Fig. 1G). We found that the number of metastatic lesions was actually higher for the SOX4^KO^ organoids (Fig. 1H). However, these metastatic foci covered a smaller proportion of the lungs than in the control tumors (Fig. 1I). In accordance, the size per metastatic lesion was significantly larger in control tumors than in SOX4 ^KO^ tumors (Fig. 1J). Similar analyses using H&E staining confirmed these findings (Supplementary Fig. 2A–D). This suggests that SOX4 is not required for the initial seeding, but instead supports metastatic outgrowth. Furthermore, in line with our previous findings that SOX4 can mediate tumor angiogenesis [17], these SOX4-deficient tumors were less vascularized than control tumors (Supplementary Fig. 2E–F).

Taken together, these data show that loss of SOX4 leads to a strong impairment of primary tumor growth and metastatic outgrowth in a luminal breast cancer model.

### Loss of SOX4 does not induce a reduction of EMT in PyMT organoids and tumors

**Fig. 2:**
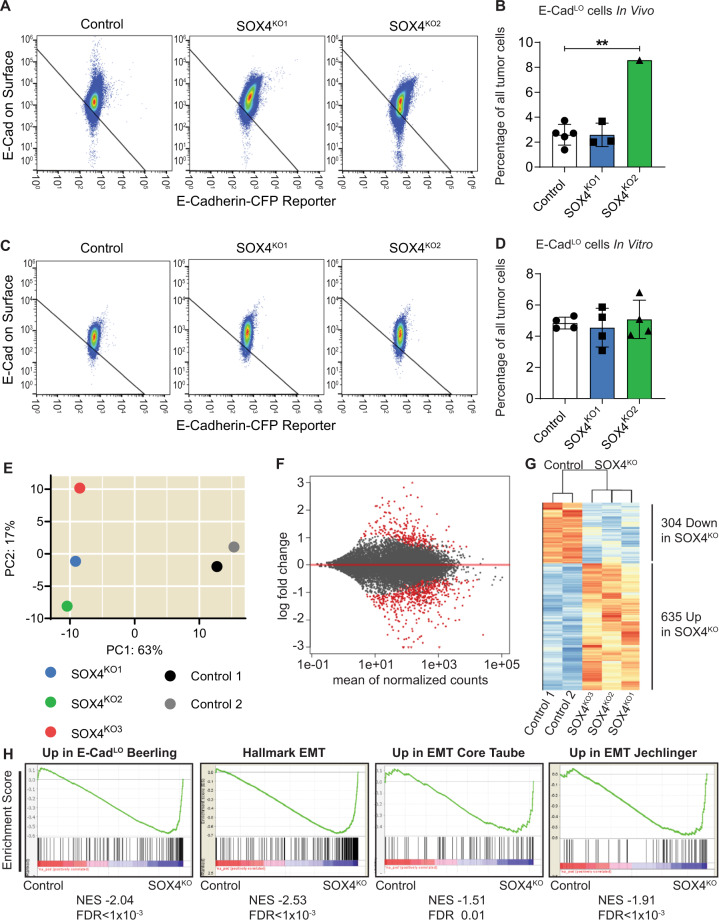
Loss of SOX4 does not induce a loss of EMT. **A** FACS plots to determine E-Cad^LO^ cells by FACS in tumors. Tumors were isolated and subjected to a FACS protocol as described in materials and methods. FACS plots show E-cadherin antibody staining on Y-axis and E-cadherin CFP-reporter expression on X-axis. E-Cad^LO^ cells are found in the left-bottom gate. Plots are representative pictures for each of the three groups (control, SOX4^KO1^, SOX4^KO2^). **B** Quantification of E-Cad^LO^ cells for control and SOX4^KO^ tumors shown as percentage of parental population (single cells). Data is represented as average ± SD. ANOVA using Dunnett test for multiple comparisons indicated non-significant differences (*p* > 0.05) of SOX4^KO1^ and **p < 0.01 for SOX4^KO2^. **C** FACS plots of E-Cad^LO^ cells in organoids in vitro in similar analysis as 2(A). Plots for each of the three groups (control, SOX4^KO1^, SOX4^KO2^) are representative pictures corresponding to one experiment. Experiment was performed four times. **D** Quantification of E-Cad^LO^ cells for control and SOX4^KO^ organoids in vitro shown as percentage of parental population (all single tumor cells). Data is represented as average ± SD. ANOVA using Dunnett test for multiple comparisons indicated non-significant differences (*p* > 0.05) of SOX4^KO^ lines compared to control. **E** Principal Component Analysis of three SOX4^KO^ organoid lines and two control organoid lines. **F** Differential gene expression analysis between control and SOX4^KO^ organoids lines. Gray dots indicate genes; red dots indicate significant genes (adjusted *p*-value < 0.1). **G** Heatmap showing differentially expressed genes between control and SOX4^KO^ organoids. 304 genes are significantly downregulated and 635 genes are significantly upregulated in SOX4^KO^ organoids. **H** Gene-set enrichment analysis (GSEA) representing the enrichment of four different EMT gene sets ([8], Hallmark EMT in GSEA [42, 43]) in the bulk RNA-seq expression dataset.

Tumors from PyMT organoid transplantations contain a small population of E-cad^LO^ cells [8] (Supplementary Fig. 3A), which exhibit a typical EMT expression profile [8, 9] (Supplementary Fig. 3B). To interrogate whether SOX4 regulates EMT in this PyMT model we performed flow cytometry analyses on primary tumors to quantify the E-cad^LO^ cells. Unexpectedly, we did not find consistent reductions in E-cad^LO^ cells in the SOX4^KO^ lines (Fig. 2A–B, Supplementary Fig. 3C–D). We also found no significant differences in E-cad^LO^ cells after growth in vitro (Fig. 2C–D, Supplementary Fig. 3E–F). Furthermore, in Western blots there was no clear increase in E-cadherin expression, while the mesenchymal marker N-cadherin was modestly upregulated in SOX4^KO^ organoids (Supplementary Fig. 3G). Moreover, mRNA expression of EMT markers that were previously found to be SOX4 transcriptional targets in mammary epithelial cells [21], was not consistently affected (Supplementary Fig. 3H).

To explore whether SOX4 affects mesenchymal gene expression in an unbiased fashion, we performed bulk RNA-sequencing on organoids in vitro. Besides the three lines used previously we chose an additional control and SOX4^KO^ organoid line (Supplementary Fig. 1A). Principal component analysis of the bulk RNA-sequencing data showed that SOX4^KO^ organoids are clearly distinct from control organoids (Fig. 2E). We found more than 900 genes that were significantly differentially expressed between control and SOX4^KO^ organoid lines (Fig. 2F–G, Supplementary Table 1). We compiled a ranked list of genes based on their difference in expression between control and SOX4^KO^ organoids. Gene set enrichment analysis (GSEA) was used to determine whether previously published genes involved in EMT were enriched in either control or SOX4^KO^ organoids. These analyses showed that mesenchymal genes were enriched in SOX4^KO^ organoids (Fig. 2H). Taken together these data suggest that, in contrast to what might be expected, SOX4-deletion leads to an induction of mesenchymal gene expression in the PyMT-organoid model.

### SOX4 is required to maintain a fetal mammary stem cell gene expression program in mammary tumors

**Fig. 3:**
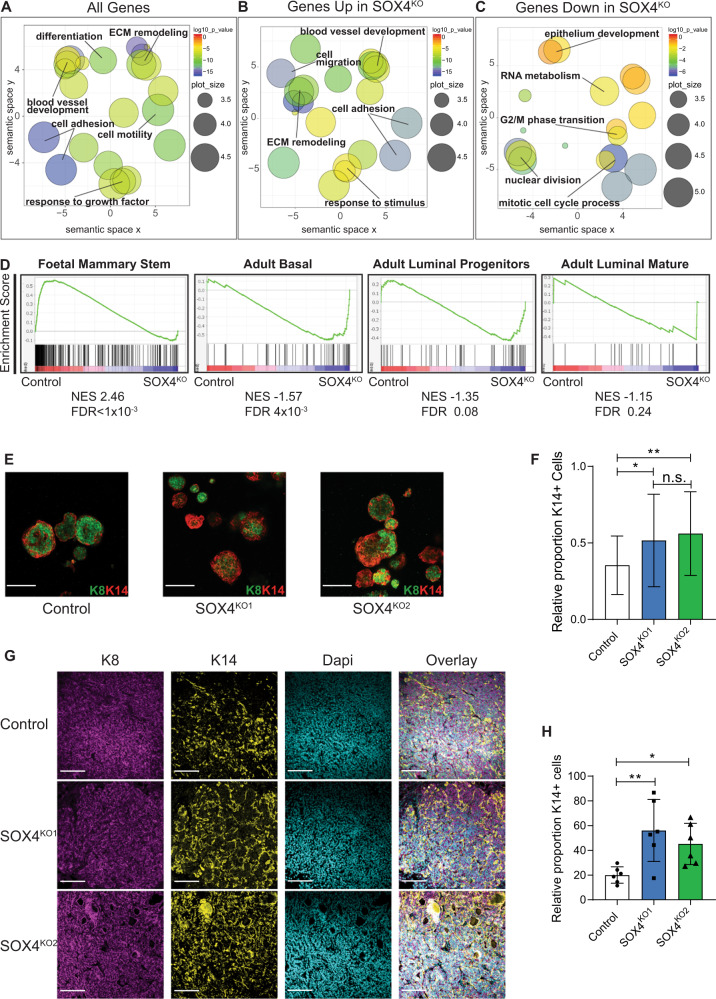
Loss of SOX4 is associated with an increase in mammary differentiation. **A** GO-term analysis of all differentially expressed genes. GO-terms are visualized by REVIGO to summarize similar GO-terms [44]. **B** GO-term analysis of 635 significantly upregulated genes in SOX4^KO^ organoids. **C** GO-term analysis of 304 significantly downregulated genes in SOX4^KO^ organoids. **D** GSEA comparing genes differentially expressed in SOX4^KO^ organoids to gene sets specific for fetal mammary stem cells, adult mammary basal cells, adult mammary luminal progenitors and adult mammary luminal mature cells (derived from [26]). **E** Confocal images of immunostaining for luminal marker K8 (green) and basal marker K14 (red) on organoids in vitro. **F** Quantification of proportion of organoids that exhibits predominantly K14 staining. Data is represented as average ± SD. ANOVA using Dunnett test for multiple comparisons was used to calculate *p*-values (n.s. = non-significant, *p < 0.05, **p < 0.01). Scale bar is 100 µm. **G** Confocal images of immunostaining for luminal marker K8 (magenta) and basal marker K14 (yellow) and DAPI (Cyan) on paraffin sections of lung metastases. Scale bar is 100 µm. **H** Quantification of K14-positive cells as a proportion the K8-positive cells, which make up all tumor cells. Data is represented as average ± SD. ANOVA using Dunnett test for multiple comparisons was used to calculate *p*-values (*p < 0.05, **p < 0.01).

To characterize the differentially expressed genes between control and SOX4^KO^ organoids, the ToppGene portal [25] was utilized to identify significant enrichments in gene ontology. These analyses suggest that SOX4 regulates a wide variety of pro-oncogenic processes, including extracellular matrix remodeling, adhesion, blood vessel development and cell differentiation (Fig. 3A). Of these the most strongly affected biological processes were extracellular matrix remodeling and adhesion (Fig. 3A, Supplementary Fig. 4A). However, these processes were mostly associated with the genes that are upregulated in SOX4^KO^ organoids (Fig. 3A–B, Supplementary Fig. 4A). Conversely, the genes that were downregulated in SOX4^KO^ organoids were most strongly associated with cell-cycle regulation (Fig. 3C, Supplementary Fig. 4A). Motif analysis for these genes indicated that SOX binding sites are the most prevalent binding sites found in promoter regions of these genes (Supplementary Table 2), which suggests the downregulated genes in SOX4^KO^ organoids are direct targets of SOX4.

We also compared SOX4-dependent genes to gene expression profiles of other cell-types using ToppCell Atlas [25]. There was a significant association of basal mammary genes with the list of all SOX4-dependent genes (Supplementary Fig. 4B). It has recently been suggested that SOX transcription factors are of major importance to mammary differentiation [26]. We compared SOX4-dependent differentially expressed genes to gene sets for cell types of specific stages and lineages during mammary development [26]. A loss of expression of fMaSC genes was observed in the SOX4^KO^ organoids. The ‘core’ enrichment of fMaSC genes in control organoids (which are those genes that most strongly contribute to enrichment [27]) consisted of a large proportion of the fMaSC geneset (118/300), suggesting that SOX4 has a strong impact on fMaSC genes in PyMT tumor organoids (Supplementary Table 3). Conversely, we found an enrichment of basal genes in the SOX4^KO^ organoids, while luminal genes did not exhibit a significant enrichment in control or SOX4^KO^ organoids (Fig. 3D). By qRT-PCR we confirmed that expression of fMaSC genes was reduced in SOX4^KO^ organoids (Supplementary Fig. 4C). In contrast luminal markers showed variable correlation in SOX4^KO^ organoids (Supplementary Fig. 4D), while expression of basal markers was confirmed to be upregulated (Supplementary Fig. 4E). To further explore whether SOX4 is required for regulating differentiation of PyMT breast tumors we analyzed expression of the luminal marker Keratin 8 (K8) and the basal marker Keratin 14 (K14). These experiments showed that loss of SOX4 results in an increased number of K14-positive basal-like cells in organoids in vitro (Fig. 3E–F). In primary mammary tumors, the number of K14-positive cells appeared to be either very high or very low, precluding any conclusive analyses on the small number of SOX4^KO^ tumors (Supplementary Fig. 4F–G). However, in accordance with Cheung et al. [28] in lung tumors after tail vein injections, the number of K14-positive cells was on average higher than in the mammary glands. In these analyses SOX4^KO^ tumors in the lungs exhibited a higher proportion of K14-positive cells than the control tumors (Fig. 3G–H). In addition, the primary tumors and the lung tumors were histologically analyzed by H&E stainings and were classified by qualified pathologists into four categories of variable differentiation status (see materials and methods for more information). In the primary tumors we could not identify a correlation between morphology patterns and SOX4 (Supplementary Fig. 4H–I), possibly due to the low number of SOX4^KO^ tumors and the severe differences in tumor growth. In SOX4^KO^ lung tumors however, we found an increase in the number of tubular structures, which are more reminiscent of the normal mammary gland (Supplementary Fig. 4J–K). It has been been proposed that differentiated cells are required for establishing tubules in the normal mammary gland^29^ and our data supports this concept.

**Fig. 4:**
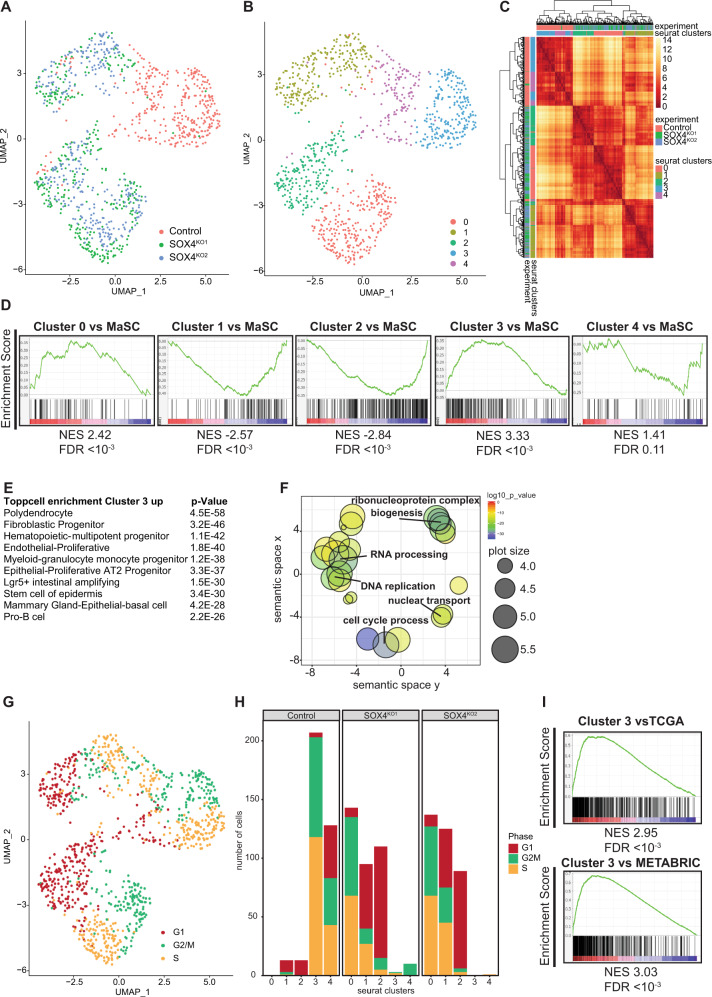
SOX4 is required for maintaining a progenitor cell cycle program. **A** UMAP projections of single cell RNA-sequencing analyses of control and SOX4^KO^ organoids color-coded for organoid line of origin. **B** UMAP projections of single cell RNA-sequencing analyses color-coded for clusters. **C** Heatmap depicting distance between clusters. **D** Gene Set Enrichment Analyses comparing genes specifically upregulated in the 5 clusters to genes specific to fMaSC [29]. **E** Top10 cell types exhibiting significant enrichment for genes specifically upregulated in cluster 3 (compared to all other clusters) as determined by Toppcell Atlas. **F** GO-term analysis of genes upregulated in cluster 3 compared to other clusters. **G** UMAP projections of single cell RNA-sequencing analyses color-coded for cell cycle phase classification. **H** Cell cycle phase classification quantified per cluster. **I** GSEA comparing genes ranked on their degree of co-expression with SOX4 in the TCGA and METABRIC datasets (Supplementary Table 6) to genes upregulated in cluster 3.

These data suggest that SOX4 impairs differentiation of mammary tumors at the cellular and tissue level. We then aimed to understand how SOX4 affects differentiation at single-cell resolution. To this end we performed single cell RNA-sequencing on organoids in vitro. Data dimensionality reduction using Seurat showed that control organoids clearly clustered separately from the SOX4^KO^ organoids (Fig. 4A–B). The control organoids were mostly found in 2 relatively similar clusters (clusters 3 and 4). The SOX4^KO^ organoids however were found in 3 clusters, of which cluster 1 was more distantly related to clusters 0 and 2 (Fig. 4B–C). For each cluster, sets of significantly up and downregulated genes were compiled (Supplementary Table 4). These cluster-specific gene sets showed that there was a strong correlation between the bulk sequencing and the single cell sequencing experiments (Supplementary Fig. 5A), strengthening the validity of both datasets.

The cluster-specific genes were compared to signature gene sets for adult basal, adult luminal and fetal mammary stem cells that were previously determined by single-cell sequencing [29]. In accordance with the bulk sequencing, we found that cluster 3, which contained the majority of the control cells, exhibited strong enrichment for fMaSC genes (Fig. 4D). In contrast, the clusters 1 and 2 exhibit a negative correlation with fMaSC genes indicating that these are found in a less undifferentiated state (Fig. 4D). Indeed cluster 1 cells express many luminal genes, while specific basal genes correlated most strongly with cluster 2 (Supplementary Fig. 5B-C). Finally, Cluster 0 does exhibit enrichment for fMaSC genes (Fig. 4D), but these cells have a lower number of genes in common with the fMaSCs in comparison with cluster 3 cells indicating that cluster 0 cells are less stem-like than cluster 3 cells (Supplementary Fig. 5D-E). Taken together, these data suggest that SOX4 is required to maintain PyMT mammary tumor cells in an undifferentiated state.

### SOX4 regulates a conserved progenitor cell-cycle gene expression program

To understand whether SOX4 has a general role in regulating progenitor gene expression, we assessed which cell types exhibit significant correlation with the genes upregulated in cluster 3 using ToppCell Atlas. We found that SOX4-dependent genes were almost exclusively correlated with gene expression programs of stem/progenitor cells (Fig. 4E). Among these were polydendrocytes [30], LGR5 ^+ ^intestinal cells [31], multipotent hematopoietic cells [32, 33], skin stem cells [34] and pro-B-cells [35]. These are specific cell types that have previously been shown to rely on SOX4-activity for their maintenance [30–35]. In contrast clusters 1, 2 and 4 were more associated with differentiated cell types (Supplementary Table 5). To understand the consequences of this progenitor gene expression program we performed GO-term analysis of cluster 3 genes. This showed that these genes were associated with cell cycle progression, DNA biosynthesis, RNA processing and ribosome biogenesis (Fig. 4F, Supplementary Fig. 6A). These processes are all essential for cycling of (tumor) cells and this indicates that SOX4 is essential to keeping the cells in a state that is primed to maintain active cycling. Unbiased comparison to Hallmark gene sets in GSEA showed strongest enrichment of genes involved in G2/M progression and for target genes of E2F and MYC which are well-known for their role in cell cycling and cancer. This was specific to the SOX4-dependent cluster 3, while clusters 1 and 2 exhibited negative enrichment for such genes (Supplementary Fig. 6B–K). To further investigate the cell cycle-related gene expression of these organoids, we calculated expression scores of S- and G2/M-specific genes, and assigned cell cycle phase predictions to each cell. We observed that the more differentiated clusters 1 and 2 contained very low numbers of cells with a cycling gene expression profile (Fig. 4G–H). Therefore, the proportion of cycling cells was clearly decreased in SOX4^KO^ organoids (Fig. 4H).

To interrogate whether these findings recapitulated the role of SOX4 in human mammary tumors, we used the cbioportal resource [36, 37]. We compiled ranked lists of genes based on their co-expression with SOX4 in human breast cancers in the TCGA and METABRIC studies [38, 39] (Supplementary Table 6). These ranked lists were compared to the cluster 3 genes. This showed a significant positive correlation of genes in cluster 3 with the genes that were co-expressed with SOX4 in human cancers (Fig. 4I).

In accordance the genes that were co-expressed with SOX4 in the TCGA and METABRIC studies showed enrichment for GO-terms (Supplementary Fig. 6A) and Hallmark datasets (Supplementary Fig. 6L–M) associated with cell cycle progression. These were identical to the GO-terms and Hallmark datasets that were found to be enriched for in cluster 3 genes. Finally, to test the specificity of these findings we also assembled lists of genes co-expressed with SOX10 and TWIST in METABRIC (Supplementary Table 6). We performed similar analyses for SOX10 [26], as an alternative SOX transcription factor, and Twist, as an EMT transcription factor, to exclude the possibility that this approach would simply yield identical results for any SOX- or for any EMT transcription factor. For SOX10 and TWIST the co-expressed genes did not show strongest association to cell-cycle related hallmark datasets (Supplementary Fig. 6N–O), indicating that our findings were specific to SOX4.

Taken together, these data suggest that SOX4 is required for maintenance of a progenitor-like gene expression program that stimulates cell cycle progression in mammary tumors.

### SOX4 regulates tumor growth in a cell-autonomous manner

**Fig. 5:**
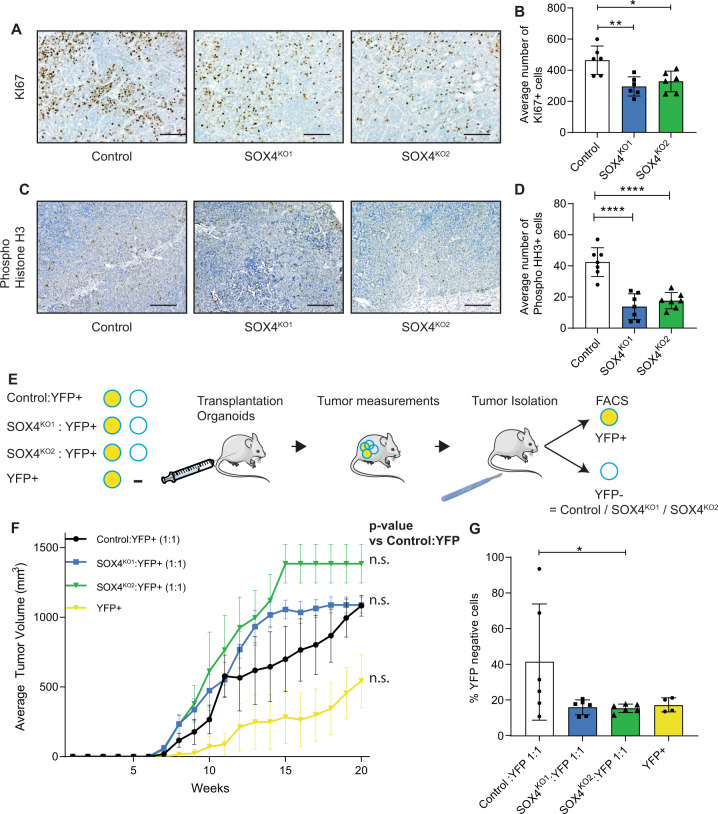
SOX4 is required for tumor proliferation in a cell-autonomous manner. **A** Ki67 staining on paraffin sections of lung metastases. Scale bar is 100 µm. **B** Quantification of Ki67^+^-cells in control and SOX4^KO^ tumors in lungs. Data is represented as average ± SD. ANOVA using Dunnett test for multiple comparisons was used to calculate *p*-values (*p < 0.05, **p < 0.01). **C** Phospho-Histone H3 (Ser10) staining on paraffin sections of lung metastases. Scale bar is 100 µm. **D** Quantification of phospho-Histone H3-positive cells in control and SOX4^KO^ tumors in lungs. Data is represented as average ± SD. ANOVA using Dunnett test for multiple comparisons was used to calculate *p*-values (****p-value < 0.0001). **E** Schematic representation of experimental setup for cell competition assay. Organoid mixtures were transplanted orthotopically in mammary fat pads of recipient mice. Mixtures consisted of 50% YFP + organoids and 50% of indicated organoids (control, SOX4^KO1^, SOX4^KO2^) or unmixed YFP + organoids. Tumor growth was measured over time. Mice were sacrificed when tumor volume reached 1000 mm^3^ and tumors were subjected to FACS to determine the percentage of YFP + and YFP- cells. **F** Growth curves for tumors after mammary transplantation of indicated mixtures of organoids. Data are represented as mean tumor volume (mm^3^). *P*-values were determined to be non-significant (> 0.05) by the “compare growth curves” method [41]. **G** Quantification of YFP negative cells (% of all tumor cells) as determined by flow cytometry for YFP on tumors.

The lack of growth of SOX4^KO^ tumors and the single cell RNA-sequencing data suggest that SOX4 regulates cycling of tumor cells. Downregulation of cell cycle genes was confirmed by qRT-PCR (Supplementary Fig. 7A). However, we did not find any significant differences in cell cycle phase by Propidium Iodide staining nor in the number of proliferative Ki67 positive cells on organoids in vitro (Supplementary Fig. 7B–E). We hypothesized that the differences in cell-cycle related gene expression would only have a more relevant functional effect in vivo. Indeed, SOX4^KO^ tumors showed reduced Ki67 staining in primary mammary tumors and in metastatic lung tumors after tail vein injections (Fig. 5A–B; Supplementary Fig. 7F–G). In addition, tumors were stained for phospho-Histone H3 (Ser10), which marks cells that are in G2-M phase transition. While Ki67-positive cells were highly abundant and found throughout the tumors, phospho-Histone H3 positive cells were much less frequently found and mostly present at the periphery of tumors. Quantification of phospho-Histone H3 expression showed that SOX4^KO^ tumors, both in the mammary gland and in the lungs, exhibit a lower number of cells in G2-M phase (Fig. 5C–D; Supplementary Fig. 7H–I). These findings coupled to the RNA-sequencing analyses suggest that SOX4^KO^ cells have a lower expression of cell cycle regulatory genes, which results in reduced tumor growth due to reduction of proliferative cycling cells.

Since SOX4 regulates cell cycling, cells with high SOX4 expression may out-compete cells with low SOX4 expression. To test this we compared the growth of SOX4-proficient and SOX4^KO^ cells in the same experimental animal. We used an independent organoid line (YFP+ organoids) which was derived from a *MMTV-PyMT; MMTV-Cre; R26R-YFP; E-cad-mCF*P mouse model [8] and which could be distinguished from control/SOX4^KO^ organoids by flow cytometry. We mixed single cell suspensions of control/SOX4^KO^ organoids with YFP+ organoids (after independent growth in vitro). These mixtures were transplanted into mammary fat pads (Fig. 5E–F) and we found that, for the control:YFP+ mixtures, the control organoids comprised 41% of all analyzed tumor cells (Fig. 5G) indicating that control and YFP+ organoids exhibit comparable growth potentials in vivo. Tumors that consisted of mixtures of SOX4^KO^ cells with YFP+ cells showed no significant differences in growth to the mixtures of control cells with YFP+ cells (Fig. 5F, Supplementary Fig. 7J). However, flow cytometry analyses showed that the vast majority of cells within tumors derived of mixtures of SOX4^KO^ cells and YFP+ cells were in fact YFP+ cells (Fig. 5G). These data indicate that SOX4-deficient cells are severely compromised in tumor growth, even in the presence of SOX4-proficient cells.

Taken together these data suggest that SOX4 affects tumor growth by maintaining a stem/progenitor gene program which regulates proliferative capacity in a cell-autonomous manner.

## Discussion

In this study RNA-sequencing data demonstrated that SOX4 is required to regulate a fetal mammary stem cell gene program which is abundant in cell cycle genes. In a recent study Dravis et al. performed transcriptomic and epigenetic analyses on fMaSC. In support of our study they found that fMaSCs exhibit multi-lineage potential and that many fMaSC genes contain SOX-binding sites [26]. Here, we show that SOX4 mediates breast tumor-progression through maintenance of fMaSC gene expression. In addition, we show that the fMaSC gene program impairs differentiation and is essential to cell cycling. Functionally, the genes regulated by SOX4 are involved in control of cell division. In accordance, both primary and secondary tumors are strongly impaired in growth and exhibit reduced proliferation due to loss of SOX4. Moreover, in SOX4^KO^ organoids we found an increase in cells exhibiting more differentiated gene expression patterns, particularly of adult basal cells. An increase in the number of K14-positive basal-like cells was also found in SOX4^KO^ lung tumors. Terminally differentiated cells within tumors have often been associated with an inability to cycle and to fuel tumor growth [10–12]. In our experiments we find indeed that SOX4^KO^ tumors exhibit a reduced number of Ki67+ proliferative cells and a lower number of phospho-HistoneH3+ cells indicating a lower number of cells in G2M phase transition. Together these data suggest that loss of SOX4 results in a higher proportion of differentiated post-mitotic cells.

Impairing differentiation reflects a general role for SOX4 in maintaining progenitor identity since SOX4-dependent genes were also enriched for genes specific to other progenitor cell types. Compellingly, we found association to the very progenitor cell types that SOX4 has been shown to be essential for in normal developmental and homeostatic processes such as polydendrocytes, multipotent lymphoid progenitors, pro-B-cells, LGR5+ intestinal stem cells and skin stem cells [30–35]. Until now, it has remained elusive which mechanisms underlie the activity of SOX4 in such a broad array of cells. Here we show that SOX4 impairs differentiation and enforces cell cycle gene expression programs that may be relevant to all of these diverse cell types. Our findings suggest that SOX4 expression in mammary tumors leads to re-activation of such developmental gene programs. Considering the broad deregulation of SOX4 expression in cancer this may occur in other tumor types as well. We propose that SOX4-mediated cell-cycle regulation is a major underlying aspect in human breast cancer development. This is supported by the findings that the SOX4-dependent genes in PyMT tumor-organoids strongly overlap with genes correlating with SOX4 in human mammary tumors in the TCGA and METABRIC studies and the previously published correlation of SOX4 and mitotic index in breast cancer patients [17].

The degree to which SOX4 can regulate cell cycling clearly depends on the niche as we find larger differences in proliferation in the breast niche than in the lung niche. Moreover in vitro loss of SOX4 does not affect proliferation or cell cycle regulation, presumably because the continuous supply of oxygen, growth factors and nutrients masks inherent differences in the ability to proliferate and cycle. Our data shows that in vivo loss of SOX4 does result in a reduced number of proliferative cells and the number of cells in G2M phase. We speculate that the more challenging in vivo environment selects for cells with high expression of cell cycle related genes to grow out, which are strongly reduced in SOX4^KO^ tumors.

Our data suggests that SOX4 regulates mammary tumor growth in the PyMT model independently of promoting EMT. In contrast, ourselves and others have previously shown that SOX4 positively regulates EMT in mammary epithelial cells [21–23]. In the current study we found that a loss of SOX4 in PyMT breast tumor organoids is not associated with a corresponding loss of E-cad^LO^ cells. RNA-sequencing even suggests that loss of SOX4 directs PyMT organoids to a more mesenchymal state. While these findings appear at first to be counterintuitive, they may be explained through the context-dependent nature of SOX4 DNA-binding and experimental differences between the various studies. We have previously shown that SOX4 relies on a pre-existing epigenome, signaling pathways and protein levels to determine its target genes [16, 17]. The luminal ductal PyMT organoid in vivo model is distinct from the in vitro cell lines that have previously been used to demonstrate that SOX4 regulates EMT [17, 21–23]. These cell lines were either untransformed mammary epithelial cells such as HMLE [17], MCF10A [22] and NMuMG [23] or more mesenchymal tumor cells (MDA-MB-231, MCF10A-RAS, Py2T) [17, 22, 23]. It is worth noting that only a very limited number of cells undergo full EMT in the PyMT model [8], but many tumor cells are found in an intermediate state, which better reflects human breast tumors than most cell lines [8, 9, 40]. In tumors from a basal or claudin-low subtype the epigenetic and signaling context may be more permissive for SOX4 to induce EMT.

Our study further supports the current dogma that EMT and tumor stemness are uncoupled [4, 8, 24]. SOX4^KO^ organoids exhibit impaired tumor growth in primary tumors but this is independent of EMT. Similarly, SOX4^KO^ organoids were found to exhibit decreased potential for forming metastatic colonies in the lung after injection in the tail vein. Our findings suggest that the fMaSC gene program is a driving factor for mammary tumor proliferation and acts distinctly from EMT. It should be interesting to interrogate whether the re-expression of proliferative fMaSC genes is a general mechanism by which mesenchymal cells acquire the potential to grow out into (epithelial) tumors.

The central concept emerging from this study proposes that SOX4 maintains cells in an undifferentiated state and promotes cell cycle progression. This function is most likely critical for progression of both solid and hematological cancers, and mirrors its ability to maintain progenitor pools in embryonic development. Future work should be aimed at developing methodologies to interfere with SOX4-functionality, thereby stimulating differentiation of tumor cells to a post-mitotic state.

## Materials and methods

### Cell Culture of PyMT tumor organoids

Organoids were derived as described previously [8]. Organoids were cultured in drops of 50 μL matrigel. The organoids were cultured in DMEM/F12 + Glutamax + HEPES + Penicillin-Streptomycin with 2% B27-Supplement and 12.5 ng/mL Recombinant Human Fibroblast Growth Factor-basic.

### Organoid transplantation and mastectomy in mice

Control, SOX4^KO1^ and SOX4^KO2^ organoids were harvested and made into single cell suspensions using trypsinization. Per mouse 250,000 cells were injected in 100 μL PBS. Tumor growth was measured using a caliper. If the mouse developed a tumor of 1000 mm^3^, a mastectomy was performed. All mice were sacrificed 3 weeks after mastectomy or at 16 weeks after transplantation. Metastases were quantified by eye by two independent researchers after careful inspection of the lungs.

## Supplementary information

Supplementary Information and Figures

Supplementary Table 1

Supplementary Table 2

Supplementary Table 3

Supplementary Table 4

Supplementary Table 5

Supplementary Table 6

## Data Availability

RNA-sequencing data generated in this study has been deposited to GEO and is available under GSE153190.
