# Supplementary material for: Regulation of a progenitor gene program by SOX4 is essential for mammary tumor proliferation
Source: Oncogene. 2021 Sep 28;40(45):6343–53. doi: 10.1038/s41388-021-02004-z (PMC8585668; doi:10.1038/s41388-021-02004-z)

Supplementary Information

Supplementary materials and methods

Key resources table

Reagent or resource	Source	Identifier
Experimental Models: Cell Lines		
Organoid line Control 1	Derived from a tumor from a MMTV-PyMT; MMTV-Cre; Ecadherin-mCFP GEMM.	N/A
Organoid line Control 2	Derived from a tumor from a MMTV-PyMT; MMTV-Cre; Ecadherin-mCFP GEMM	N/A
Organoid line $SOX4^{KO1}$	Derived from a tumor from a MMTV-PyMT; MMTV-Cre; Ecadherin-mCFP GEMM	N/A
Organoid line $SOX4^{KO2}$	Derived from a tumor from a MMTV-PyMT; MMTV-Cre; Ecadherin-mCFP GEMM	N/A
Organoid line $SOX4^{KO3}$	Derived from a tumor from a MMTV-PyMT; MMTV-Cre; Ecadherin-mCFP GEMM	N/A
Organoid line YFP+	Derived from a tumor from a *MMTV-PyMT; MMTV-Cre; R26R-YFP; E-cad-mCFP* GEMM	N/A
Experimental Models: Mouse models		
NOD-scid $Il2ry^{null}B2m^{null}$	Jackson Laboratory	Stock No:010636
MMTV-PyMT; MMTV-Cre; Ecadherin-mCFP GEMM	MMTV-PyMT – Jackson Laboratory<br>MMTV-Cre – Jackson Laboratory<br>E-Cadherin-mCFP –Hans Clevers Lab	Stock No: 002374<br>Stock No: 003553<br>N/A
MMTV-PyMT; MMTV-Cre; R26R-YFP; E-cad-mCFP GEMM	MMTV-PyMT – Jackson Laboratory<br>MMTV-Cre – Jackson Laboratory<br>E-Cadherin-mCFP –Hans Clevers Lab<br>R26R-YFP – Jacqueline Deschamps Lab	Stock No: 002374<br>Stock No: 003553<br>N/A<br>N/A
Antibodies		
E-Cadherin for western blot	BD transduction	Cat#610182
N-Cadherin for western blot	BD transduction	Cat#610921
SOX4 for western blot	Diagenode	Cat#CS-129-100
Tubulin for western blot	Sigma-Aldrich	Cat#T5168
E-cad-eFluor660 for flow cytometry	eBioscience	Cat#DECMA-1

GFP for IHC	Abcam	Cat#ab6673
Ki67 for IHC	Thermo Scientific	Cat# RM-9106-S0
PE-anti human Ki-67 antibody for flow cytometry	Biolegend	Cat# 350503
anti-Phospho Histone H3 (Ser10) for IHC	Millipore	Cat# 06-570
Keratin, type II/ Cytokeratin 8 antibody	Developmental Studies Hybridoma Bank	Cat# TROMA-1
Keratin 14 antibody	Biolegend	Cat# 905301
Endomucin for IHC	Abcam	Cat# ab106100
Chemicals		
Prestoblue cell viability reagent	Thermofisher	Cat#A13261
DNase I	Roche	Cat#4716728001
Isoflurane	Pharmachemie BV, Haarlem, Netherlands	Cat# 45.112.110
Histopaque-1077	Sigma	Cat#10771
DMEM/F12 + GlutaMAX	Invitrogen Life Technologies	Cat#10565018
TH Liberase	Roche	Cat#5401151001
Collagenase A	Roche	Cat# 10103578001
Matrigel	Corning	Cat# 356231
B27	Thermo Fisher Scientific	Cat# 17504044
Recombinant Human Fibroblast Growth Factor-basic	Gibco	Cat# 2016613
**Oligonucleotides**		
**Target (QPCR)**	**Sequence**	
**EMT genes**		
E-Cadherin Fw	tgtctaccaaagtgacgctga	
E-Cadherin Rv	ctctgggttggattcagagg	
Vimentin Fw	aagaacacccgcaccaac	
Vimentin Rv	gtaagttggcaaagcggtca	
N-Cadherin Fw	gccatcatcgctatccttct	
N-Cadherin Rv	ccgtttcatccataccacaa	
Zeb1 Fw	tggcaagacaacgtgaaaga	
Zeb1 Rv	aactgggaaaatgcatctgg	

MMP2 Fw	gcttctgtcctgaccaaggat
MMP2 Rv	ggaagttcttggtgtaggtgtagat
Fibronectin Fw	tgccctgaagaacaatcaga
Fibronectin Rv	aaccagttggggaagctcat
B2M Fw	cgtgcgtgacatcaaagaga
B2M Rv	cgctcgttgccaatagtgat
GAPDH Fw	aatgtgtccgtcgtggatct
GAPDH Rv	aatgtgtccgtcgtggatct
**fMaSC genes**	
Ccnb1 Fw	gcgctgaaaattcttgacaac
Ccnb1 Rv	ttcttagccaggtgctgcat
Igsf9 Fw	agggggaagctacctcagtc
Igsf9 Rv	catagttctcatccacagaggtgt
Col1a1 Fw	ccgctggtcaagatggtc
Col1a1 Rv	ctccagcctttccaggttct
Ndc80 Fw	ccgatcacactcccctctt
Ndc80 Rv	ttcgtcttcacccattatgtttt
Plk1 Fw	ccatcccaccaaggttttc
Plk1 Rv	agggggttctccacacct
Kif11 Fw	tgaagaaagctgtaaagcagga
Kif11 Rv	ttttcctctctggtgtcatacct
Mcm5 Fw	ctttgtctggcaatctgtcg
Mcm5 Rv	aatgcggctcagcatctc
Dtl Fw	aagtgggaagacgtcaccag
Dtl Rv	agaagtcatcttgagtctttctacga
**Luminal genes**	
Esr1 Fw	ctctgggcgacattcttctc
Esr1 Rv	ggaccagaggtacatccatttc
Erbb2 Fw	aacagctcggagacctgcta
Erbb2 Rv	gtagtgggcacaagcctca
Kit Fw	gatctgctctgcgtcctgtt
Kit Rv	cttgcagatggctgagacg
Elf5 Fw	gactgtcacagccgaacaag
Elf5 Rv	ccaggatgccacagttctct
Krt18 Fw	agatgacaccaacatcacaagg
Krt18 Rv	cttccagaccttggacttcct
K8 Fw	agttcgcctccttcattgac
K8 Rv	ccacttggtctccagcatct
**Basal genes**	
Smtn Fw	atctgattctcccgtggttg
Smtn Rv	gtggtgctgatgagggaag
Krt14 Fw	atcgaggacctgaagagcaa
Krt14 Rv	tcgatctgcaggaggacatt
Itgb1 Fw	tgcttctaaaattgagatcaggag
Itgb1 Rv	tccataaggtagtagagatcaataggg
Krt17 Fw	aggagctggcctacctgaa
Krt17 Rv	acctggcctctcagagcat
**Cell cycle genes**	
Myb Fw	gagtggaccaccgttactgaa
Myb Rv	tttcccgatttctcagttgg
Gsta4 Fw	cccctgtactgtccgacttc
Gsta4 Rv	aggaatgttgctgattcttgtct
Rrm1 Fw	tgtattcgggccactggtag

Rrm1 Rv	attggcacaaggccattaga
Fubp1 Fw	ggcttgggaagaatactacaagaa
Fubp1 Rv	tataatccggctgaccacct
Mex3a Fw	acaccacggagtgcgttc
Mex3a Rv	gttggttttggccctcaga
Mcm6 Fw	acctgtaccacaatctctgcac
Mcm6 Rv	caccgcgttttacttcatca
Ncaph Fw	aagccgatgggaagtgtg
Ncaph Rv	ccagtcgtgctgcattcat
Cenpe Fw	cgcctcccagtctgttgt
Cenpe Rv	tcactgtcccgtgtggaag
**gRNAs**	**Sequence**
gRNA1 (SOX4$^{KO1/3}$) Fw	caccgatcgagcggcgcaagatca
gRNA1 (SOX4$^{KO1/3}$) Rv	aaactgatcttgcgccgctcgatc
gRNA2 (SOX4^{KO2}) Fw	caccgcgccttgccgcccgtcgacg
gRNA2 (SOX4^{KO2}) Rv	aaaccgtcgacgggcggcaaggcgc

Generation of SOX4^{KO} organoids

gRNA sequences to target mouse *SOX4* were designed using the online resource on crispr.mit.edu from the Zhang Lab (currently not available anymore). 2 independent gRNAs were designed and cloned as DNA-oligo's into the pSpCas9(BB)-2A-puro vector(1). Successful cloning was verified by sequencing and plasmids were electroporated in PyMT organoids using a NEPA21 Electroporation system according to the protocol described by Fujii et al(2). 48 hours after electroporation puromycin was added to the medium. 72 hours later medium was replaced by medium without puromycin. Organoids were grown up as bulk, trypsinized and distributed as single cells in a concentration of 1000 cells /50 ul matrigel drop. Single organoids were picked and expanded. Genomic DNA of these single-clone organoid cultures was isolated using a DNeasy DNA Isolation Kit (Qiagen). For each gRNA we selected clones bearing a mutation that was predicted to lead to a frameshift in protein translation. To ensure that the organoid cultures consisted of a single clone the genomic DNA surrounding the gene-edited sequence in the *SOX4* locus was cloned in a PJET cloning vector for the selected clones. The resulting constructs were grown up in DH5α cells and 10 colonies were picked for each culture. All SOX4^{KO} organoid lines used in this manuscript were shown to contain a bi-allelic homozygous indel. Control organoids were generated by following this procedure with pSpCas9(BB)-2A-puro, SOX4^{KO1} and SOX4^{KO3} organoids were generated with pSpCas9(BB)-2A-puro containing gRNA 1. SOX4^{KO2} organoids were generated with pSpCas9(BB)-2A-puro containing gRNA 2.

Mice

Non-obese diabetic SCID IL-2 receptor gamma chain knockout (NSG) mice (Jackson Laboratories) were housed under IVC conditions. All mice used were females of 8-20 weeks old. Mice received food and water *ad libitum*. All experiments were carried out in accordance with the guidelines of the Animal Welfare Committee of the NKI, The Netherlands.

Western blot

For each condition 4 wells of organoids were collected. After removal of matrigel by centrifugation (800RPM for 4min, 4 degrees) the cells were lysed in 200 ul Laemmli buffer [0.12 mol/L Tris-HCL (pH 6.8), 4% SDS and 20% glycerol]. Protein concentration was determined using Lowry protein assay. Equal amounts of sample (30 µg) were analyzed by Sodium dodecyl sulfate polyacrylamide gel electrophoresis (SDS-PAGE) and electrophoretically transferred to polyvinylidene difluoride membrane (Milipore, Bedford, MA). The membranes were blocked with 10% milk protein in TBST (0.3% Tween, 10 mM Tris pH8

and 150 mM NaCl in H_2O) and probed with primary antibodies. After washing with TBST membranes were incubated with secondary antibodies. Membranes were subsequently washed with TBST and detection was done using ECL and detected with a BioRad Chemidoc Touch Imaging system.

Proliferation assays

Proliferation assays were performed using Prestoblue (Thermo Fisher Scientific). Organoids were plated out as single cells after trypsin digestion in 10 uL of 1:1 matrigel/medium drops in a 96 Well plate. Cells were seeded in a concentration of 5.000 cells per well. Proliferation was measured at indicated timepoints by adding 10uL PrestoBlue reagent for 1 hour at 37 degrees. Each measured time point and condition was measured in triplicate per experiment. Empty wells containing only Matrigel and medium but without cells were used as background controls. Fluorescence was measured using Fluoroskan Ascent FL (Thermo Scientific) by excitation on 544 nm and measuring an emission of 570 nm.

Outgrowth from single cells

Organoids were trypsinized to single cells and diluted into the following concentrations: 20.000, 50.000 or 100.000 cells /ml. For each condition 3 drops of 50 ul were plated out. Organoids were cultured for a week and counted. For each condition representative pictures were analyzed by ImageJ to determine average organoid size.

Quantification of mRNA expression (qRT-PCR)

mRNA was extracted from organoids using the RNeasy Isolation Kit (Qiagen) according to the manufacturer’s protocol. 1000 ng of total RNA was reverse transcribed using iScript cDNA synthesis kit (Bio-Rad) to obtain cDNA. cDNA samples were amplified using SYBR green supermix (Bio-Rad) using a LightCycler 96 system instrument (Roche) according to the manufacturer's protocol. To quantify the data, the comparative Ct method was used. Relative fold change was defined as 2-ΔΔCt and β2-microglobulin was used as a reference gene.

Tail vein injections

Organoids were harvested and made into single cell suspensions using trypsin. The organoids were diluted in PBS and 250.000 cells were injected in the tail vein of recipient NSG-mice. All mice were sacrificed after 10 weeks.

Transplantation of ‘mixed’ organoids

Control, $SOX4^{KO1}$, $SOX4^{KO2}$ and YFP+ organoids were cultured independently and harvested and made into single cells using trypsinization. The cells were counted, diluted in PBS and mixed in the right ratio’s. Per mouse 250.000 cells were injected in 100 ul PBS except for the YFP+ only group which received only 125.000 cells. NSG mice were sedated using isoflurane inhalation anesthesia (1.5% to 2% isoflurane/O2 mixture) and the organoids were injected beneath the 4th nipple. If the mouse developed a tumor of 1000 mm^3 the mice were sacrificed. Metastatic lesions in the lungs were counted by two independent researchers.

Flow cytometry tumors

Tumors were collected and minced manually on ice using sterile scalpels. The tumor mass was digested in PBS supplemented with 25 µg/ml DNase I (Roche) and 5 Wünsch units TH Liberase /ml (Roche) at 37 °C for 35 min, followed by mashing through a 70-µm filter (BD Falcon) while adding DMEM/F12 + GlutaMAX (GIBCO, Invitrogen Life Technologies) supplemented with 5% (v/v) foetal bovine serum (Sigma), 100 µg/ml streptomycin, and 100 U/ml penicillin (Invitrogen Life Technologies) and 25 µg/ml DNase. After spin down (4 min at 500 RCF at RT), the pellet was resuspended in 6 ml 5 mM EDTA/PBS, after which a Ficoll gradient (Histopaque-1077, Sigma) was used to select for live cells (30 min at 400 RCF at RT, break 1). Cells were washed once in 5 mM EDTA/PBS and centrifuged (4 min at 500 RCF at RT) before proceeding with antibody labeling. The cells were blocked in 80% FACS buffer

(5 mM EDTA in PBS supplemented with 5% foetal calf serum)/20% serum mix (50/50 normal goat serum (monx10961, Monosan) for 10 min on ice before labeling with E-cad-eFluor660 (DECMA-1, eBioscience) for 1 hr at 4 degrees. Cells were washed with FACS buffer. Then cells were spun down (4 min, 500 RCF at RT) and supernatant was discarded. Cells were resuspended in FACS buffer and analyzed on a CytoFLEX flow cytometer (Beckman Coulter).

Flow cytometry organoids
For each condition 3 wells of organoids were collected and incubated in PBS supplemented with 25 µg/ml DNase I (Roche) and 5 Wünsch units TH Liberase /ml (Roche) at 37 °C for 35 min. Subsequently the cells were further mechanically separated using an insulin needle until a single cell suspension was obtained. Cells were washed once in 5 mM EDTA/PBS and centrifuged (4 min at 500 RCF at RT) before proceeding with antibody labeling. The cells were blocked in 80% FACS buffer (5 mM EDTA in PBS supplemented with 5% foetal calf serum)/20% serum mix (50/50 normal goat serum (monx10961, Monosan) for 10 min on ice before labeling with E-cad-eFluor660 (DECMA-1, eBioscience), Propidium Iodide (P4864, Sigma-Aldrich) or PE-anti human Ki-67 antibody (350503, Biolegend) for 1 hr at 4 degrees. Cells were washed with FACS buffer. Then cells were spun down (4 min, 500 RCF at RT) and supernatant was discarded. Cells were resuspended in FACS buffer and analyzed on a CytoFLEX flow cytometer (Beckman Coulter).

Bulk RNA sequencing
Organoids were plated out as single cells at equal concentrations and were grown for 4 days until they were lysed. RNA was isolated using the RNeasy Isolation kit (Qiagen) according to the manufacturer's protocol. Purified RNA was subsequently repurified using mRNA-ONLY Eukaryotic mRNA Isolation Kit (Epicentre (Illumina, Inc.), Madison, WI, USA).
Sequencing libraries were made using the Rapid Directional RNA-Seq Kit (NEXTflex) and sequenced on Illumina NextSeq500 to produce single-end 75 base long reads (Utrecht Sequencing Facility). Reads were aligned to the mouse reference genome NCBIM37 mm9 using STAR version2.4.2a. Read groups were added to the compressed binary version of the sequence alignment file (*.bam) files using Picard's "AddOrReplaceReadGroups" tool (v1.98). The bam files were sorted with Sambamba v0.4.5, and transcript abundances were quantified with HTSeq-count version 0.6.1p117 using the union mode. Subsequently, reads per kilobase of transcript per million reads sequenced were calculated with edgeR's rpkm() function. Differentially expressed genes in the transcriptome data were identified using the DESeq. 2 package with standard settings(3). PCA was performed in R, using the plotPCA() command. Reads were normalized for sequencing depth and rlog transformed. HOMER software was used for motif discovery, peak annotation and the generation of histograms(4).

Gene Ontology Analyses
GO term analysis was performed using the Toppgene portal (https://toppgene.cchmc.org/).
Genes that were identified as significantly up- or downregulated in $SOX4^{KO}$ organoids compared to control organoids were entered into the Toppfun section, which was used to analyze functional enrichment in gene ontology and in similarities to cell types (using the Toppcell Atlas module). GO-terms were visualized by REVIGO (http://revigo.irb.hr/) to cluster similar GO-terms.

Single Cell RNA-sequencing
Control, $SOX4^{KO1}$ and $SOX4^{KO2}$ organoids were trypsinized to obtain single cell suspensions. Single cells were then FACS-sorted into 384-well plates containing 100 nL of barcoded CEL-Seq2 primers and dNTPs. RNA isolation, cDNA library preparations, Illumina sequencing, and read alignment to the mouse genome (NCBIM37 mm9) were then performed by Single Cell Discoveries as described before(5). The mapped reads were counted using STAR(6) and then further processed in Rstudio (version 1.2.5019, Rstudio Team, 2019) and R (version 3.6.1, R Core Team, 2019) using the Seurat package version 3.1(7). Only cells in which less than 2%

of the UMI counts mapped to mitochondrial genes, and at least 1000 genes were expressed, passed the quality control. Next, data where normalized and scaled with the SCTransform method(8). The first twenty principle components where used to perform clustering analysis using the FindNeighbours and FindClusters functions in Seurat. Cells were visualized as diffusion maps (UMAPs). Cell cycle phase scores were calculated using Seurat's built-in scoring function(9). This function calculates z scores based on expression of a panel of S-phase genes and a panel of G2/M-specific genes. These scores are then used to predict discrete cell cycle phase classifications (G1, S, G2M) to each cell. Heatmaps were created by plotting the Manhattan distances between each individual cell with the R package Pheatmap version 1.0.12.

Gene Set Enrichment Analysis
Pre-ranked analysis was performed with the GSEA software probing for enrichment of genes belonging to the sets referred to in the main text and figure legends, or to Hallmark datasets in the GSEA software.

Co-Expressed genes of SOX4, SOX10 and Twist
Cbioportal (https://www.cbioportal.org/) was used and queried by gene of interest in TCGA and/or METABRIC studies. Co-expression section was used to generate a list of co-expressed genes which were used as a ranked list based on spearman's correlation coefficient.

Immunohistochemistry
H&E staining & Morphological analyses
Three paraffin sections at different depths in the tissue (each separated by 100um) were stained by H&E using the following procedure. Paraffin sections of 4 µm were immersed in xylene and subsequently immersed in different percentages of ethanol (100%, 95%, 70% and 50%). After rehydration hematoxalin and eosin staining was performed. Whole-mount slides were scanned using a Hamamatsu Nanozoomer scanner and analyzed by two qualified pathologists (AdB and SDN) using the NDP.view2 software. Metastatic lesions were quantified by measuring number of metastatic lesions per slide. In addition surface area of tumor tissue was quantified and compared to surface area of healthy (lung) tissue to obtain percentage tissue covered by tumor.

The morphological patterns were assessed in a semi-quantitative way. To this end two independent pathologists scored the entire tissue slides after H&E staining and scanning into four categories based on the classification by (10). The contribution of each pattern to the overall tumor mass (only the non-necrotic part of the tumor was considered) was evaluated by eye and expressed in percent. The patterns were: tubular, solid, cord-like, and mixed. The tubular pattern was characterized by areas showing tubules formation (spaces lined by a row of neoplastic cells). The solid pattern consisted of dense sheet of cells without further identifiable pattern of organization. The cord-like pattern (which is here considered as a type of solid pattern) comprised cords/trabecules (of variable thickness) of neoplastic cells, the cord being lined by a fibrovascular stroma. Finally, the mixed pattern consisted of variable combination of the luminal and cord-like patterns, without any clearly identifiable predominant component. If an area is composed of different patterns but one of them represents almost the entirety of the area, then the area is classified according to this pattern. Tubular and mixed patterns are more reminiscent of a differentiated healthy mammary gland than the solid and the cord-like patterns.

GFP staining of paraffin sections
GFP staining can be used to stain CFP-positive tumors due to high sequence similarity of GFP and CFP. Paraffin sections of 4 µm were firstly immersed in xylene for 10 minutes at room temperature and subsequently immersed in different percentages of ethanol (100%, 95%, 70% and 50%) for approximately 2 minutes each step. Tissue was rinsed in deionized water and

boiled in citrate buffer (10mM C6H5Na3O7.2H2O pH 6.0) for 20 minutes. Slides were put aside to cool down and thereafter washed in PBS. After that, slides were blocked in PO-block buffer (95 ml PBS with 5 ml H_2O_2 (37%)) for 15 minutes at room temperature. Washing step in PBS was performed and second block in PBS containing 2.5% bovine serum albumin (Sigma) for 30 minutes at room temperature. Subsequently, tissue was incubated with anti-GFP antibody (Abcam, ab6673) in PBS containing 0.5% bovine serum albumin (Sigma) for 3 days at 4 degrees. After primary antibody incubation, tissues were washed in PBS and incubated with rabbit anti-goat antibody (BIO-RAD, STAR194) for 1 hour at room temperature. After that, samples were washed in PBS and incubated with HRP-labeled anti-rabbit (ImmunoLogic, DPVB110HRP) for 30 minutes at room temperature. Tissues were washed in PBS and incubated with DAB peroxidase (vector laboratories, SK-4100) for 5 minutes at room temperature. Washing steps in demiwater were followed and tissues were incubated with hematoxyline counterstaining for 15 sec at room temperature. Slides where washed in running tapwater for 10 min. Lastly, samples where air-dried and subsequently mounted with pertex. Tissues were analyzed with an inverted bright field microscope at 10 and 40 magnification to morphologically identify metastasis. Metastatic lesions were quantified by measuring number of metastatic lesions per slide. In addition surface area of tumor tissue was quantified and compared to surface area of healthy (lung) tissue to obtain percentage tissue covered by tumor.

K8 and K14 staining on tissue sections

Formalin-fixed paraffin-embedded breast and lung tissue sections (4 µm) from mice were deparaffinized, followed by antigen retrieval using Tris-EDTA buffer for 15 min (96°C) and washing step with 1x Tris-buffered saline (TBS). Sections were blocked with 10% Normal goat serum in TBST (0.1% Tween) for 1 h at room temperature. overnight using the following dilutions: K8 (TROMA, DSHB - 1:50), K14 (Biolegend - 1:300). Tissues were then washed four times (TBST, 10 min each), incubated with secondary Alexa-fluor-488/546/647-conjugated goat anti-rat, -rabbit, and -chicken antibodies at 1:500 dilutions, together with DAPI (1 ug/mL), for 1h at RT and further washed four times (TBST, 10 min each). Sections were embedded in IMMU-MOUNT (ThermoScientific) and imaged using the Zeiss LSM 880 20x objective (NA-0,75)

K8 and K14 staining on organoids

For immunostaining, MMTVPYMT organoids embedded in matrigel were fixed with 4% paraformaldehyde (PFA; Sigma-Aldrich) for 10 minutes at room temperature and washed 3 x with 1x PBS. Fixed samples were blocked using 10% normal goat serum (Gibco) in 0.3% Triton-X (Sigma-Aldrich) iin 1x PBS for 1h. Primary antibody, diluted in antibody buffer(in 0.3% Triton-X with 1% w/v BSA in 1x PBS), was incubated at 4°C with shaking overnight, followed by at least 4 washing steps of 10-15 min each with 1x PBS. Samples were then incubated with secondary Alexa-fluor-488/546/647-conjugated goat anti-rat, -rabbit, and -chicken antibodies, together with DAPI (1 ug/mL), in antibody buffer at 4°C with shaking overnight. The following antibody concentrations were used K14 (905301, Biolegend - 1:300), K8 (TROMA-1 DSHB-1:30), Alexa-congugated secondary antibodies (1:500) secondary antibodies (1:500) goat anti-mouse IgG Alexa Fluor 488 (Invitrogen), goat-anti rabbit IgG Alexa Fluor 568 (Life Technologies) and goat anti-rat IgG Alexa Fluor 647 (Life Technologies) and DAPI 1:50 (Sigma-Aldrich). Samples were imaged using Zeiss LSM 880,using the following objectives (20×/NA 0,75).

Ki67 and Phospho Histone H3 (Ser10) staining

Paraffin sections of 4 µm were firstly immersed in xylene for 10 minutes at room temperature and subsequently immersed in different percentages of ethanol (100%, 95%, 70% and 50%) for approximately 2 minutes each step. Tissue was rinsed in deionized water and boiled in citrate buffer (10mM C6H5Na3O7.2H2O pH 6.0) for 20 minutes. Slides were put aside to cool down and thereafter washed in PBS. After that, slides were blocked in PO-block buffer (95 ml

PBS with 5 ml H_2O_2 (37%)) for 15 minutes at room temperature. Washing step in PBS was performed and second block in PBS containing 2.5% bovine serum albumin (Sigma) for 30 minutes at room temperature. Subsequently, tissue was incubated with anti-Ki67 antibody (Thermo Scientific, RM-9106-S0) or anti-phospho-Histone H3 (Ser10) (Millipore, 06-570) in PBS containing 0.5% bovine serum albumin (Sigma) for 60 min at room temperature. After primary antibody incubation, tissues were washed in PBS and incubated with poly HRP-GAM/R/R IgG (immunologic, DPVB110HRP) or Goat Anti-Rabbit IgG Antibody (H+L), Biotinylated (Vector Laboratories, BA-1000) for 30 min at room temperature. Tissues were washed in PBS and incubated with DAB peroxidase (vector laboratories, SK-4100) for 10 minutes at room temperature. Washing steps in demiwater were followed and tissues were incubated with hematoxyline counterstaining for 15 sec at room temperature. Slides where washed in running tapwater for 10 min. Lastly, samples where air-dried and subsequently mounted with pertex. Tissues were analyzed with an inverted bright field microscope at 10x magnification. To quantify positive nuclei in tumors, ImageJ software was used. Positive signal was isolated using the ImageJ colour deconvolution plug-in, followed by a fixed threshold background subtraction and automatic particle analysis, with a set circularity of 0.6.

Endomucin staining of paraffin sections

Paraffin sections of 4 µm were firstly immersed in xylene for 10 minutes at room temperature and subsequently immersed in different percentages of ethanol (100%, 95%, 70% and 50%) for approximately 2 minutes each step. Tissue was rinsed in deionized water and boiled in citrate buffer (10mM C6H5Na3O7.2H2O pH 6.0) for 20 minutes. Slides were put aside to cool down and thereafter washed in PBS. After that, slides were blocked in PO-block buffer (95 ml PBS with 5 ml H_2O_2 (37%)) for 15 minutes at room temperature. Washing step in PBS was performed and tissue was incubated with protein block* for 30 minutes at room temperature. Subsequently, tissue was incubated with anti-Endomucin antibody (Abcam, ab106100) in PBS containing 0.5% bovine serum albumin (Sigma) overnight at 4 degrees. After primary antibody incubation, tissues were washed in PBS and incubated with post primary* for 30 min at room temperature. Tissues were washed in PBS and incubated with the polymer* for 30 min at room temperature. After this tissue was washed in PBS en incubated with DAB peroxidase* for 5 minutes at room temperature. Washing steps in demiwater were followed and tissues were incubated with hematoxyline counterstaining for 15 sec at room temperature. Slides where washed in running tapwater for 10 min. Lastly, samples where air-dried and subsequently mounted with pertex. Tissues were analyzed with an inverted bright field microscope at 20x magnification. To quantify blood vessels in tumors, ImageJ software was used. Positive signal was isolated using the ImageJ colour deconvolution plug-in, followed by a fixed threshold background subtraction and automatic particle analysis, using a minimum particle size of 50 pixels. All substrates indicated with * come from the Novolink polymer detection kit (Leica, RE7290-K)

Statistical analyses

All comparisons between three groups are performed by using ANOVA using Dunnett test for multiple comparisons to calculate p-values with GraphPad Prism software. All the data were presented as mean ± SD unless stated otherwise in figure legends. All the *in vitro* experiments were performed three times independently to yield biological replicates. For animal experiments, we chose to use six animals per group, based on our previous experiences. Animals were randomly chosen to receive transplantations or injections with selected organoids. For longitudinal tumor size measurements P-values were determined by the “compare growth curves” method(11). For tumor measurements and checks for the three animal experiments described in this study the scientists performing measurements were blinded for which animal received which organoid line. For all immunostainings on paraffin sections performed in this study the scientists that performed the stainings and analyses were blind to the group allocation.

Acknowledgments:
This work was financially supported by the KWF kankerbestrijding (KWF UU 2015-7838 M.G.R., C.F.); European Research Council Grant CANCER-RECURRENCE 648804 (to J.v.R.), and the CancerGenomics.nl (Netherlands Organisation for Scientific Research) program (to J.v.R.) the Doctor Josef Steiner Foundation (to J.v.R). B.W. was financially supported by KWF kankerbestrijding (grant 11941-2018-II), ZonMW (grant 91116011) and a research infrastructure grant from Utrecht Life Sciences.

Supplementary references

1. Ran FA, Hsu PD, Wright J, Agarwala V, Scott DA, Zhang F. Genome engineering using the CRISPR-Cas9 system. Nat Protoc. 2013;
2. Fujii M, Matano M, Nanki K, Sato T. Efficient genetic engineering of human intestinal organoids using electroporation. Nat Protoc. 2015;
3. Love MI, Huber W, Anders S. Moderated estimation of fold change and dispersion for RNA-seq data with DESeq2. Genome Biol. 2014;
4. Heinz S, Benner C, Spann N, Bertolino E, Lin YC, Laslo P, et al. Simple Combinations of Lineage-Determining Transcription Factors Prime cis-Regulatory Elements Required for Macrophage and B Cell Identities. Mol Cell. 2010;38(4):576–89.
5. Muraro MJ, Dharmadhikari G, Grün D, Groen N, Dielen T, Jansen E, et al. A Single-Cell Transcriptome Atlas of the Human Pancreas. Cell Syst. 2016;
6. Dobin A, Davis CA, Schlesinger F, Drenkow J, Zaleski C, Jha S, et al. STAR: Ultrafast universal RNA-seq aligner. Bioinformatics. 2013;
7. Butler A, Hoffman P, Smibert P, Papalexi E, Satija R. Integrating single-cell transcriptomic data across different conditions, technologies, and species. Nat Biotechnol. 2018;
8. Hafemeister C, Satija R. Normalization and variance stabilization of single-cell RNA-seq data using regularized negative binomial regression. Genome Biol. 2019;
9. Tirosh I, Izar B, Prakadan SM, Wadsworth MH, Treacy D, Trombetta JJ, et al. Dissecting the multicellular ecosystem of metastatic melanoma by single-cell RNA-seq. Science (80-). 2016;
10. Cardiff RD, Anver MR, Gusterson BA, Hennighausen L, Jensen RA, Merino MJ, et al. The mammary pathology of genetically engineered mice: The consensus report and recommendations from the Annapolis meeting. Oncogene [Internet]. 2000 Feb 21 [cited 2021 Jun 24];19(8):968–88. Available from: www.nature.com/onc
11. Elso CM, Roberts LJ, Smyth GK, Thomson RJ, Baldwin TM, Foote SJ, et al. Leishmaniasis host response loci (lmr1-3) modify disease severity through a Th1/Th2-independent pathway. Genes Immun. 2004;

Supplementary Figure Legends

Supplementary Figure 1

(A) Sequencing of the targeted SOX4 locus shows 29 bp deletion in $SOX4^{KO1}$, a 44bp insertion in $SOX4^{KO2}$ and a 1 bp deletion for $SOX4^{KO3}$. The chosen indels lead to a frameshift in protein translation.
(B) Western blot for SOX4 and Tubulin .
(C) Proliferation of control organoids was compared to proliferation of $SOX4^{KO}$ organoids in a prestoblue assay (N=3).). Data is represented as average ± SD. ANOVA using Dunnett test for multiple comparisons to calculate p-values (*p<0.01)
(D) Average number of organoids grown out at seven days after splitting the organoids as single cells at a density of 20000, 50000, or 100000 cells per ml. Data indicates average number of organoids per well. Data is represented as average ± SD. ANOVA using Dunnett test for multiple comparisons indicated non-significant differences (p>0.05) of $SOX4^{KO}$ lines compared to control in all three conditions.
(E) Average organoid size corresponding to the same experiments as in Supplementary Figure 1D. Data is represented as average ± SD. ANOVA using Dunnett test for multiple comparisons indicated non-significant differences (p>0.05) of $SOX4^{KO}$ lines compared to control in all three conditions.
(F) Growth curves for individual tumors in mice transplanted with control or $SOX4^{KO}$ organoids. Data is represented as individual curves for each mouse. Growth is shown as volume (mm^3). Note that for three $SOX4^{KO1}$- and five $SOX4^{KO2}$ tumors there was no tumor growth at t = 16 weeks.
(G) Percentage of mice exhibiting tumors larger than $250mm^3$ after transplantation with control or $SOX4^{KO}$ organoids.

Supplementary Figure 2

(A) Representative images of H&E-staining on paraffin sections to identify metastases in lungs. Scale bar = 2 mm.
(B) Quantification of the number of lung macrometastases per field of view.
(C) Quantification of surface area of lungs covered by tumors expressed in percentage.
(D) Relative size per macrometastasis.
(E) Immunohistochemical stainings for the blood vessel marker endomucin on control and $SOX4^{KO}$ tumors in lungs in tail vein experiment. Scale bar is 100 µm.
(F) Quantification of number of blood vessels based on endomucin staining on control and $SOX4^{KO}$ tumors in lungs in tail vein experiment using Image-J.

Data in B-D and F is represented as average ± SD. P-values were calculated by ANOVA using Dunnett test for multiple comparisons (*p<0.05, **p<0.01).

Supplementary Figure 3

(A) Gating strategy to assess the population of $E\text{-}Cad^{LO}$ cells by flow cytometry. Triangular gate defines $E\text{-}Cad^{LO}$ cells that exhibit either low E-Cad CFP expression or internalized E-Cadherin expression (high CFP signal, but low signal for antibody staining).
(B) Quantitative RT-PCR analyses for EMT genes in $E\text{-}Cad^{LO}$ cells compared to $E\text{-}Cad^{HI}$ cells. Relative gene expression is depicted as the ratio of expression (corrected for GAPDH) in $E\text{-}Cad^{LO}$ over $E\text{-}Cad^{HI}$ cells. Data is represented as mean ± SD (p-value *<0.05, **<0.01, Students T-test).

(C) –(F) Mean Fluorescent Intensity (MFI) for the E-Cad-CFP reporter (C – primary tumors, E – organoids *in vitro*) and the E-Cadherin antibody staining (D– primary tumors, F – organoids *in vitro*). Data is represented as average MFI compared to Control. ANOVA using Dunnett test for multiple comparisons indicated non-significant differences (p>0.05) of $SOX4^{KO}$ lines compared to Control for both readouts.

(G) Western blot for E-Cadherin, N-Cadherin, SOX4 and Tubulin.

(H) Q-PCR analyses for EMT genes in control and $SOX4^{KO}$ organoids. Data is represented as mean expression ± SD, normalized to *β2M* (p-value *<0.05, **<0.01, ANOVA using Dunnett test for multiple comparisons).

Supplementary Figure 4

(A) Top 5 GO-terms for all differentially expressed genes (left) between $SOX4^{KO}$ and control organoids, genes upregulated in $SOX4^{KO}$ organoids compared to control organoids (middle) and genes downregulated in $SOX4^{KO}$ organoids compared to control organoids (right). GO-term analyses performed using Toppfun bioportal.

(B) Top 5 cell types that exhibit significant enrichment for all differentially expressed genes between $SOX4^{KO}$ and control organoids using Toppcell analysis.

(C) Q-PCR analyses for fMaSC genes in control and $SOX4^{KO}$ organoids. Data is represented as mean expression ± SD, normalized to *β2M* (p-value *<0.05, **<0.01, ANOVA using Dunnett test for multiple comparisons).

(D) Q-PCR analyses for luminal genes in control and $SOX4^{KO}$ organoids. Data is represented as mean expression ± SD, normalized to *β2M* (p-value *<0.05, **<0.01, ANOVA using Dunnett test for multiple comparisons).

(E) Q-PCR analyses for basal genes in control and $SOX4^{KO}$ organoids. Data is represented as mean expression ± SD, normalized to *β2M* (p-value *<0.05, **<0.01, ANOVA using Dunnett test for multiple comparisons).

(F) Confocal images of immunostaining for luminal marker K8 (magenta) and basal marker K14 (yellow) and DAPI (Cyan) on paraffin sections of primary tumors. Representative images show that tumors either express high or low levels of K14+ cells. Scale bar is 100 µm.

(G) Quantification of K14-positive cells as a proportion the K8-positive cells, which make up all tumor cells. Data is represented as average ± SD. ANOVA using Dunnett test for multiple comparisons was used to calculate p-values (* p<0.05).

(H) Representative images of H&E-staining on paraffin sections of primary tumors imaged at 20x magnification to identify morphological patterns described in materials and methods.

(I) Quantification of different morphological patterns based on H&E stainings on primary tumors. Data is represented as relative contribution of each pattern to the total in percentage.

(J) Representative images of H&E-staining on paraffin sections of lung tumors imaged at 20x magnification to identify morphological patterns described in materials and methods.

(K) Quantification of different morphological patterns based on H&E stainings on lung tumors. Data is represented as relative contribution of each pattern to the total in percentage.

Supplementary Figure 5

(A) Gene Set Enrichment Analyses comparing the ranked lists of differentially expressed genes between $SOX4^{KO}$ and control organoids determined by bulk RNA-sequencing to

genes upregulated in the various clusters found by single cell sequencing of control and $SOX4^{KO}$ organoids. Genes for clusters 3 and 4 that represent the majority of control cells in single-cell sequencing were also enriched in control organoids in the bulk sequencing. The other cluster-specific genesets were enriched in $SOX4^{KO}$ organoids in the bulk sequencing.

(B) Gene Set Enrichment Analyses comparing the ranked lists of genes specific for individual clusters to mammary adult luminal genes.

(C) Gene Set Enrichment Analyses comparing the ranked lists of genes specific for individual clusters to mammary adult basal genes.

(D) Venn-diagram depicting the overlap between foetal mammary stem cell genes to genes specific for cluster 0 and cluster 3.

(E) Gene expression heatmap of overlapping genes in (Supplementary Figure 5D); foetal mammary stem cell genes that are significantly elevated in clusters 0 and/or 3. Expression is calculated as z-scores from normalized RNA counts.

Supplementary Figure 6

(A) Top 5 GO-terms (Biological Processes) for the genes upregulated in cluster 3 (left), and for the top 400 genes exhibiting highest co-expression with SOX4 in METABRIC (middle) and TCGA (right).

(B) Top 3 hallmark datasets that exhibit significant enrichment for ranked list of genes upregulated in cluster 0 using Gene Set Enrichment Analysis.

(C) Top 3 hallmark datasets that exhibit significant enrichment for ranked list of genes downregulated in cluster 0 using Gene Set Enrichment Analysis.

(D) Top 3 hallmark datasets that exhibit significant enrichment for ranked list of genes upregulated in cluster 1 using Gene Set Enrichment Analysis.

(E) Top 3 hallmark datasets that exhibit significant enrichment for ranked list of genes downregulated in cluster 1 using Gene Set Enrichment Analysis.

(F) Top 3 hallmark datasets that exhibit significant enrichment for ranked list of genes upregulated in cluster 2 using Gene Set Enrichment Analysis.

(G) Top 3 hallmark datasets that exhibit significant enrichment for ranked list of genes downregulated in cluster 2 using Gene Set Enrichment Analysis.

(H) Top 3 hallmark datasets that exhibit significant enrichment for ranked list of genes upregulated in cluster 3 using Gene Set Enrichment Analysis.

(I) Top 3 hallmark datasets that exhibit significant enrichment for ranked list of genes downregulated in cluster 3 using Gene Set Enrichment Analysis.

(J) Top 3 hallmark datasets that exhibit significant enrichment for ranked list of genes upregulated in cluster 4 using Gene Set Enrichment Analysis.

(K) Top 3 hallmark datasets that exhibit significant enrichment for ranked list of genes downregulated in cluster 4 using Gene Set Enrichment Analysis.

(L) Top 3 hallmark datasets that exhibit significant enrichment for top 400 genes exhibiting highest co-expression with SOX4 in TCGA using Gene Set Enrichment Analysis.

(M) Top 3 hallmark datasets that exhibit significant enrichment for top 400 genes exhibiting highest co-expression with SOX4 in METABRIC using Gene Set Enrichment Analysis.

(N) Top 3 hallmark datasets that exhibit significant enrichment for top 400 genes exhibiting highest co-expression with SOX10 in METABRIC using Gene Set Enrichment Analysis.

(O) Top 3 hallmark datasets that exhibit significant enrichment for top 400 genes exhibiting highest co-expression with TWIST in METABRIC using Gene Set Enrichment Analysis.

Supplementary Figure 7

(A) Q-PCR analyses for cell cycle genes in control and $SOX4^{KO}$ organoids. Data is represented as mean expression ± SD, normalized to *β2M* (p-value *<0.05, **<0.01, ANOVA using Dunnett test for multiple comparisons).
(B) Representative flowcharts of Propidium Iodide stainings on organoids *in vitro*.
(C) Quantification of cell cycle distribution as calculated using the cell cycle phase Dean-Jett-Fox method in Flowjo. Data are presented as average contribution per cell cycle- phase (N=3, For each cell cycle phase ANOVA using Dunnett test for multiple comparisons which indicated non-significant differences).
(D) Representative FACS-plots of KI67 stainings on organoids *in vitro*.
(E) Quantification of $KI67^{+}$-cells in control and $SOX4^{KO}$ tumors in primary mammary tumors. Data are presented as mean ± SD (ANOVA using Dunnett test for multiple comparisons indicated non-significant differences).
(F) KI67 staining on paraffin sections of primary tumors. Scale bar is 100 µm.
(G) Quantification of $KI67^{+}$-cells in control and $SOX4^{KO}$ tumors in lungs. Data is represented as average ± SD. ANOVA using Dunnett test for multiple comparisons was used to calculate p-values (** p<0.01).
(H) Phospho-Histone H3 (Ser10) staining on paraffin sections of primary tumors. Scale bar is 100 µm
(I) Quantification of Phospho Histone H3-positive cells in control and $SOX4^{KO}$ tumors in lungs. Data is represented as average ± SD. ANOVA using Dunnett test for multiple comparisons was used to calculate p-values (* p-value<0.05).
(J) Growth curves for individual tumors in mice transplanted with mixtures of YFP+ and YFP- cells. Data is represented as individual growth curves for each mouse. Growth is shown as volume (mm^3).

Supplementary Figure 1

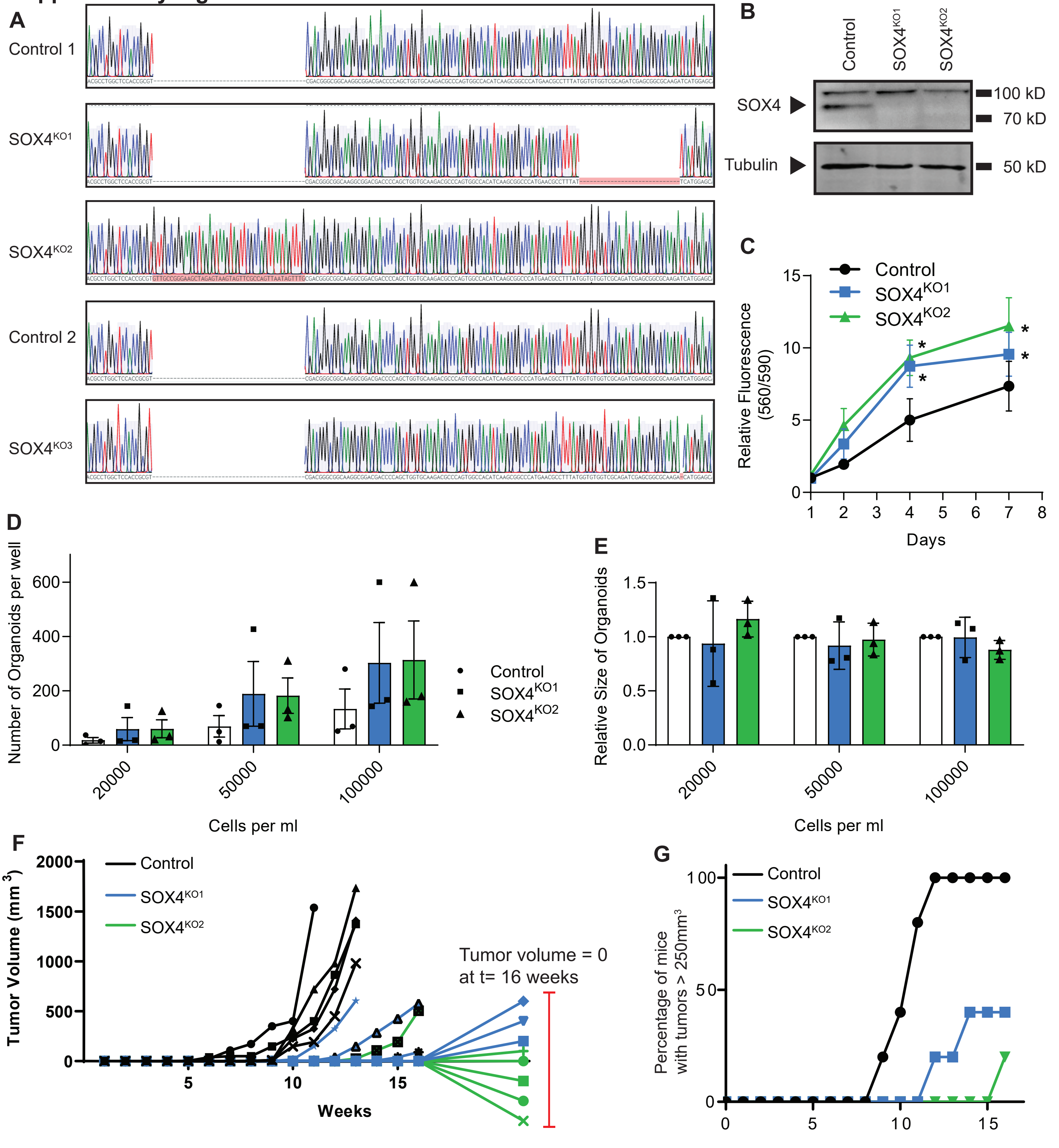

Supplementary Figure 2

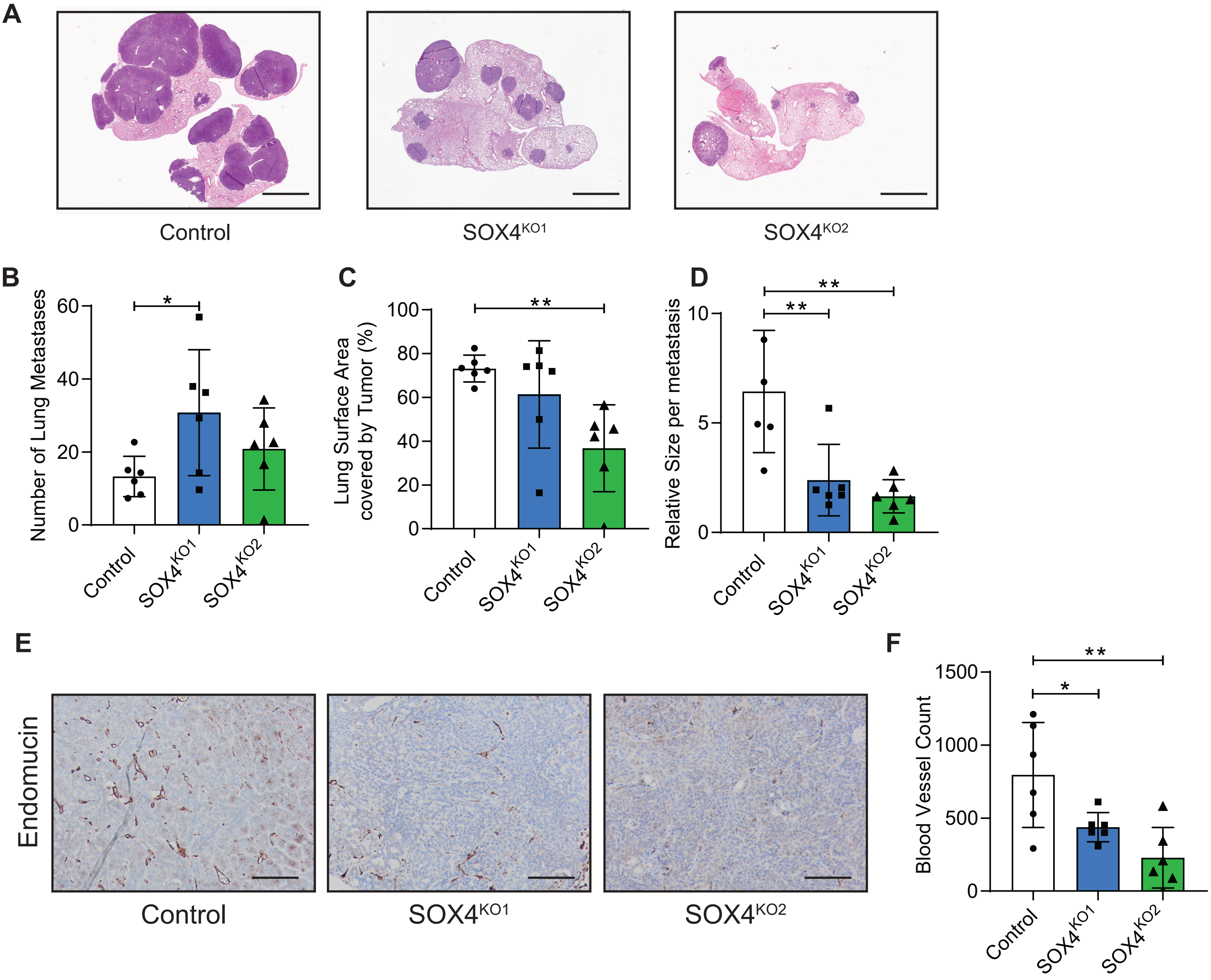

Supplementary Figure 3

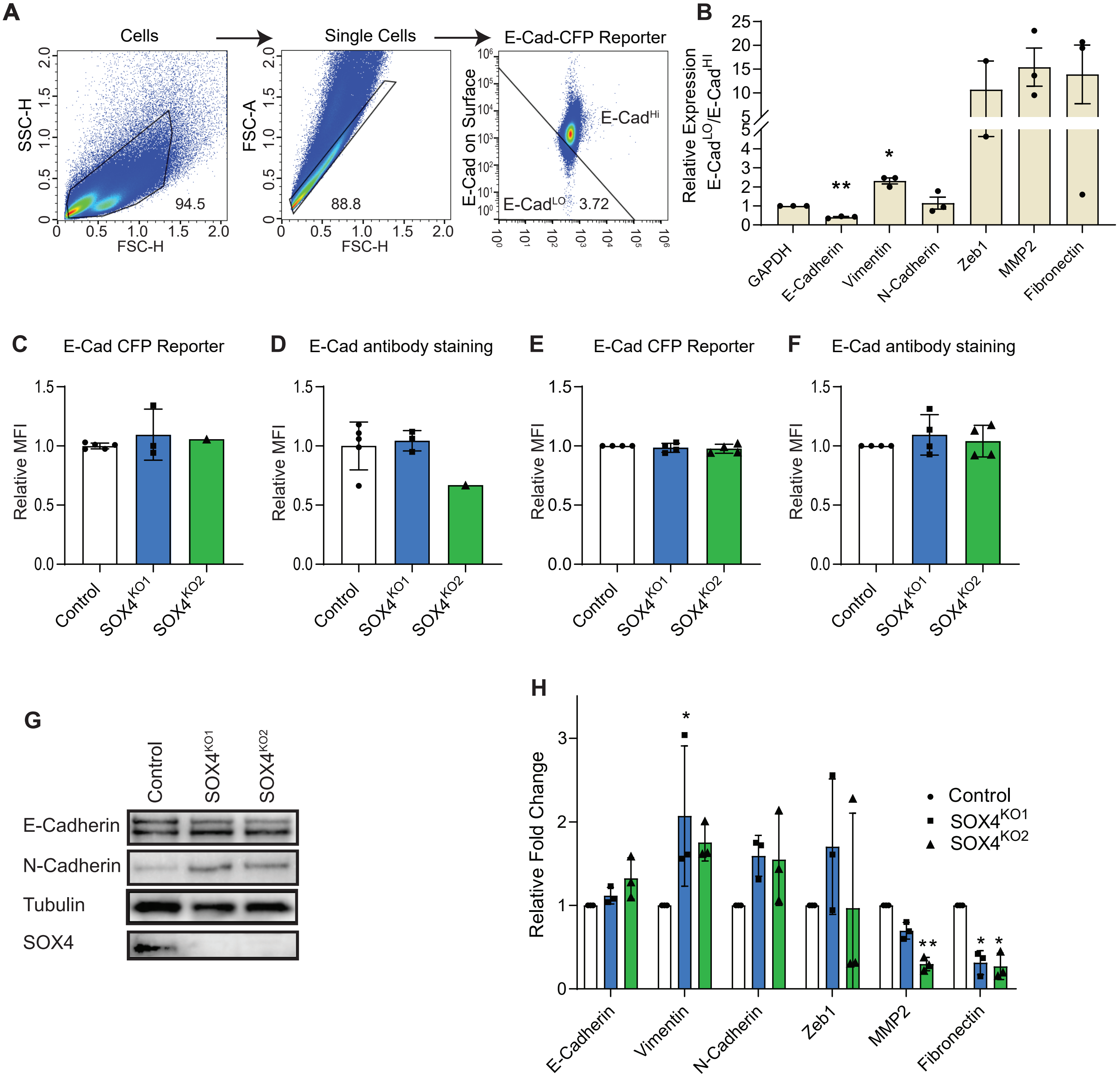

Supplementary Figure 4

A

All genes	p-value	Genes Up in SOX4^{KO}	p-value	Genes Down in SOX4^{KO}	p-value
1. Biological adhesion	8.76E-18	1. Extracellular structure organization	1.48E-19	1. Mitotic nuclear division	6.03E-7
2. Cell adhesion	1.48E-17	2. Extracellular matrix organization	3.11E-19	2. Mitotic cell cycle	6.03E-7
3. Extracellular matrix organization	1.63E-16	3. Cell migration	4.39E-16	3. Mitotic cell cycle process	6.03E-7
4. Extracellular structure organization	3.80E-16	4. Biological adhesion	4.94E-16	4. Cell division	2.74E-6
5. Animal organ morphogenesis	3.27E-15	5. Cell adhesion	1.03E-15	5. Nuclear division	5.65E-6

B

Toppcell Atlas - analyses all genes	pValue
1. Epithelial-basal cell--ts16 Mammary Gland	1.074E-24
2. Epithelial-basal cell of Epithelial	3.681E-21
3. Epithelial-basal cell--ts18 Tongue	1.696E-18
4. Endothelial cell	5.850E-18
5. Epithelial-keratinocyte stem cell	1.005E-15

C

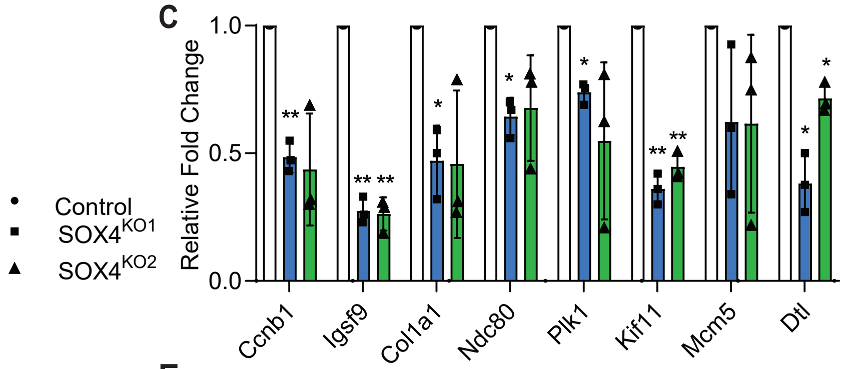

D

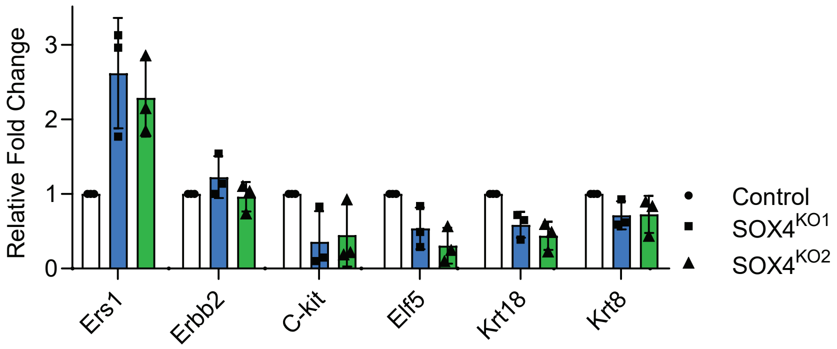

E

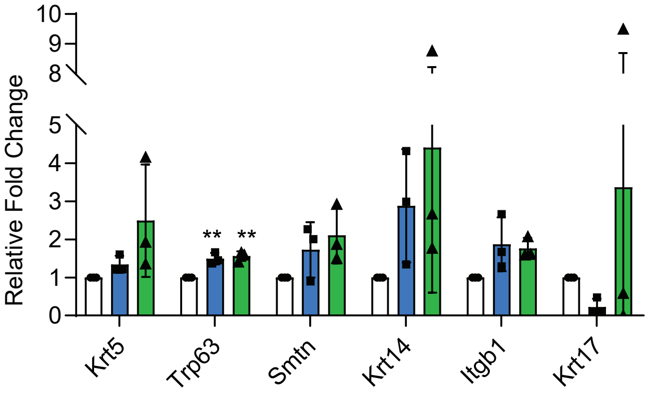

F

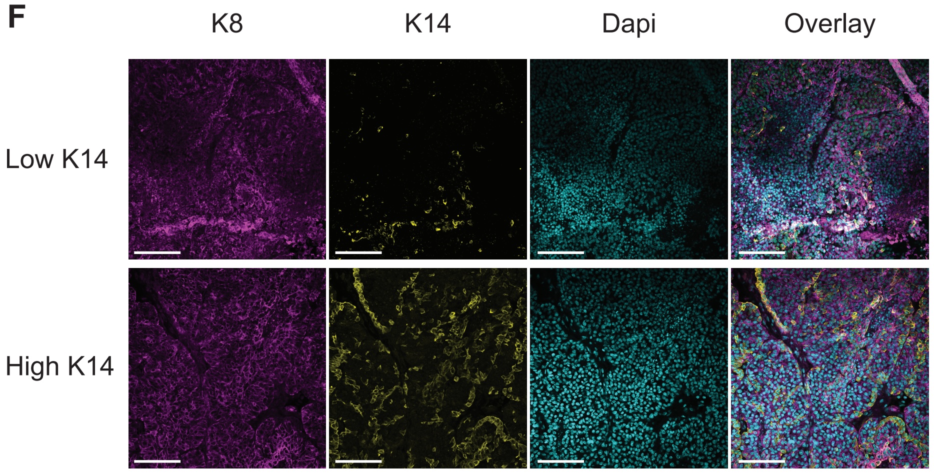

G

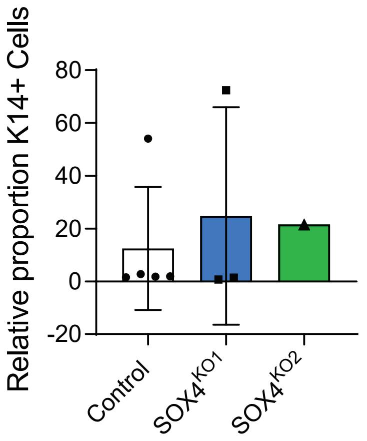

H

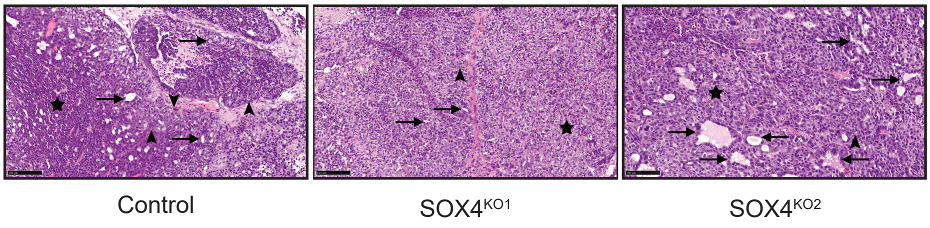

I

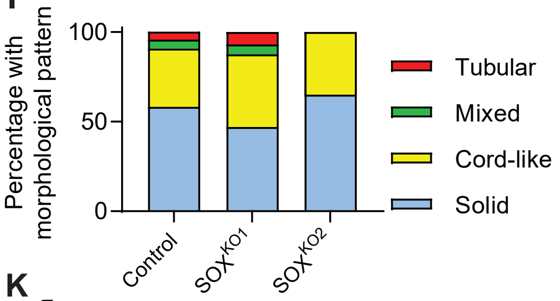

J

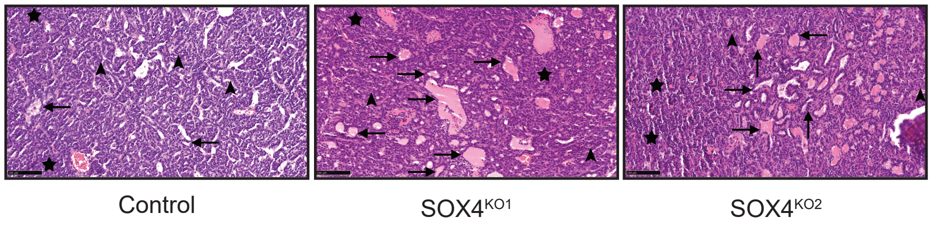

K

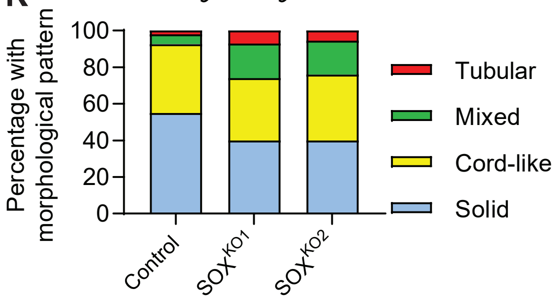

Supplementary Figure 5

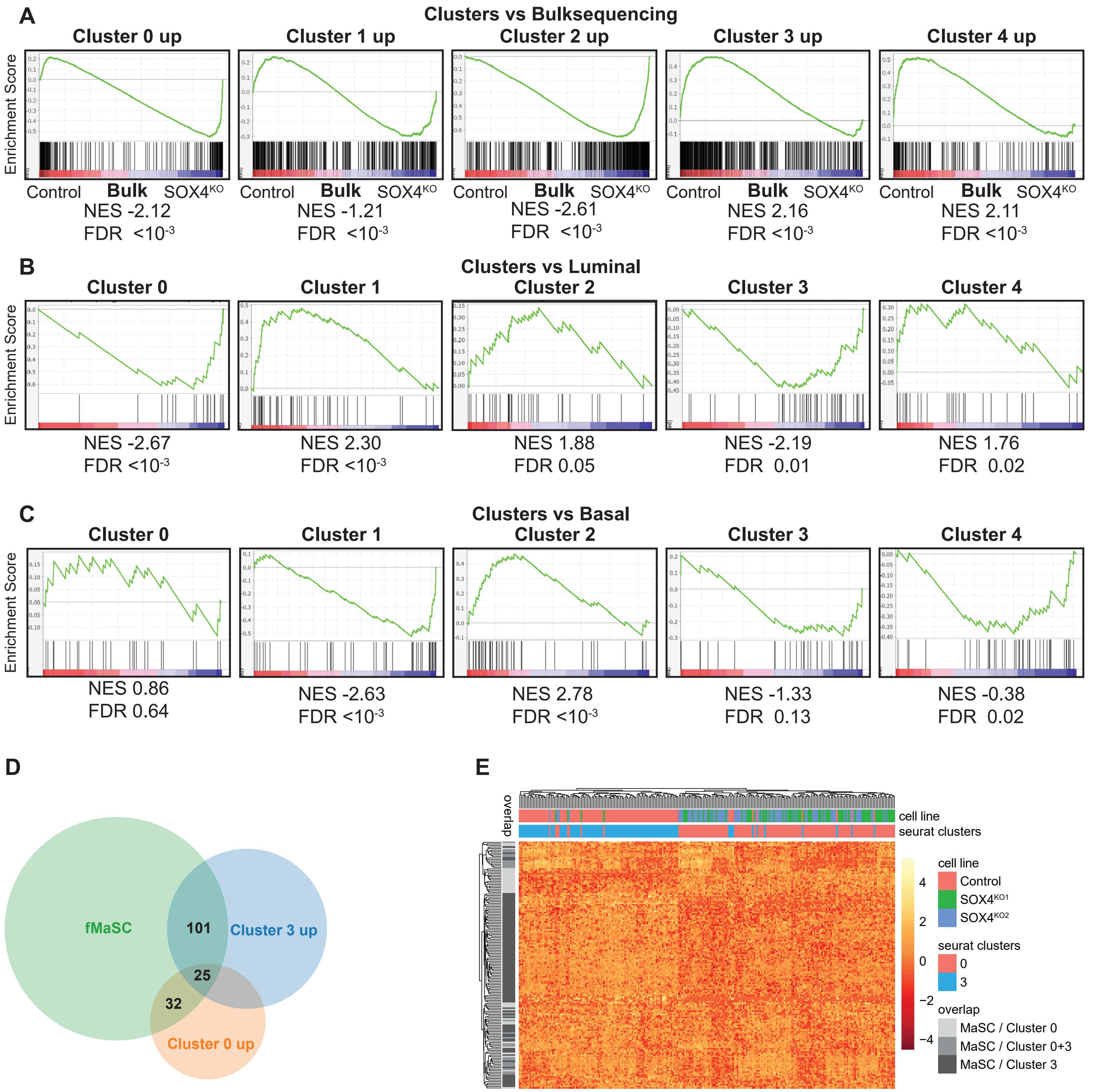

Supplementary Figure 6

A

Cluster 3		METABRIC		TCGA	
1.Mitotic cell cycle	9.87E-35	1.Organelle fission	2.80E-09	1.Nuclear division	4.58E-15
2.Mitotic cell cycle process	9.87E-35	2.Mitotic cell cycle	3.64E-09	2.Organelle fission	8.80E-15
3.Mitotic nuclear division	9.87E-35	3.Mitotic nuclear division	3.64E-09	3.Mitotic cell cycle	7.47E-14
4.Nuclear division	1.23E-30	4.Mitotic cell cycle process	3.64E-09	4.Mitotic nuclear division	7.47E-14
5.Cell cycle	1.20E-29	5.Nuclear division	5.62E-09	5.Mitotic cell cycle	7.47E-14

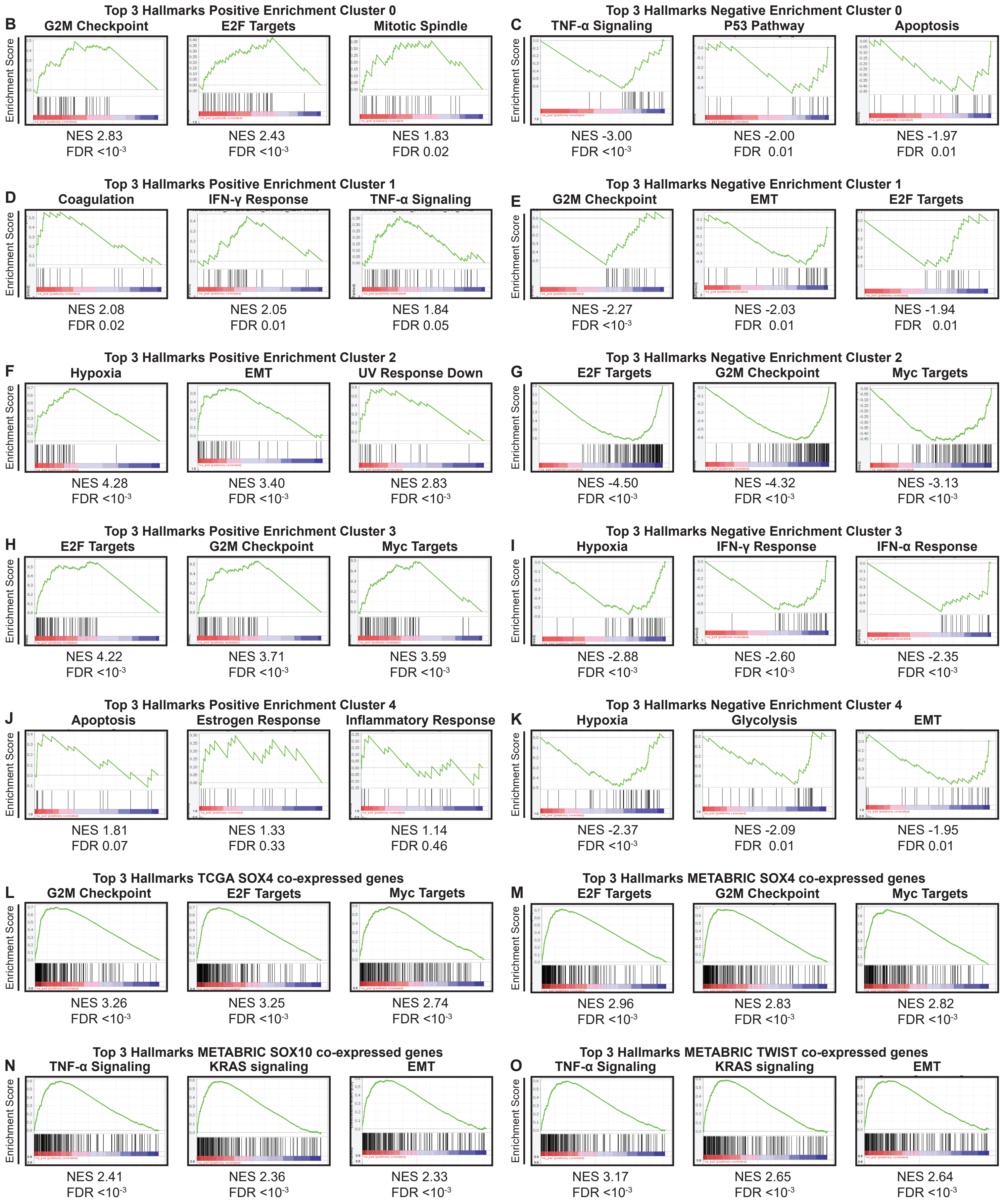

Supplementary Figure 7

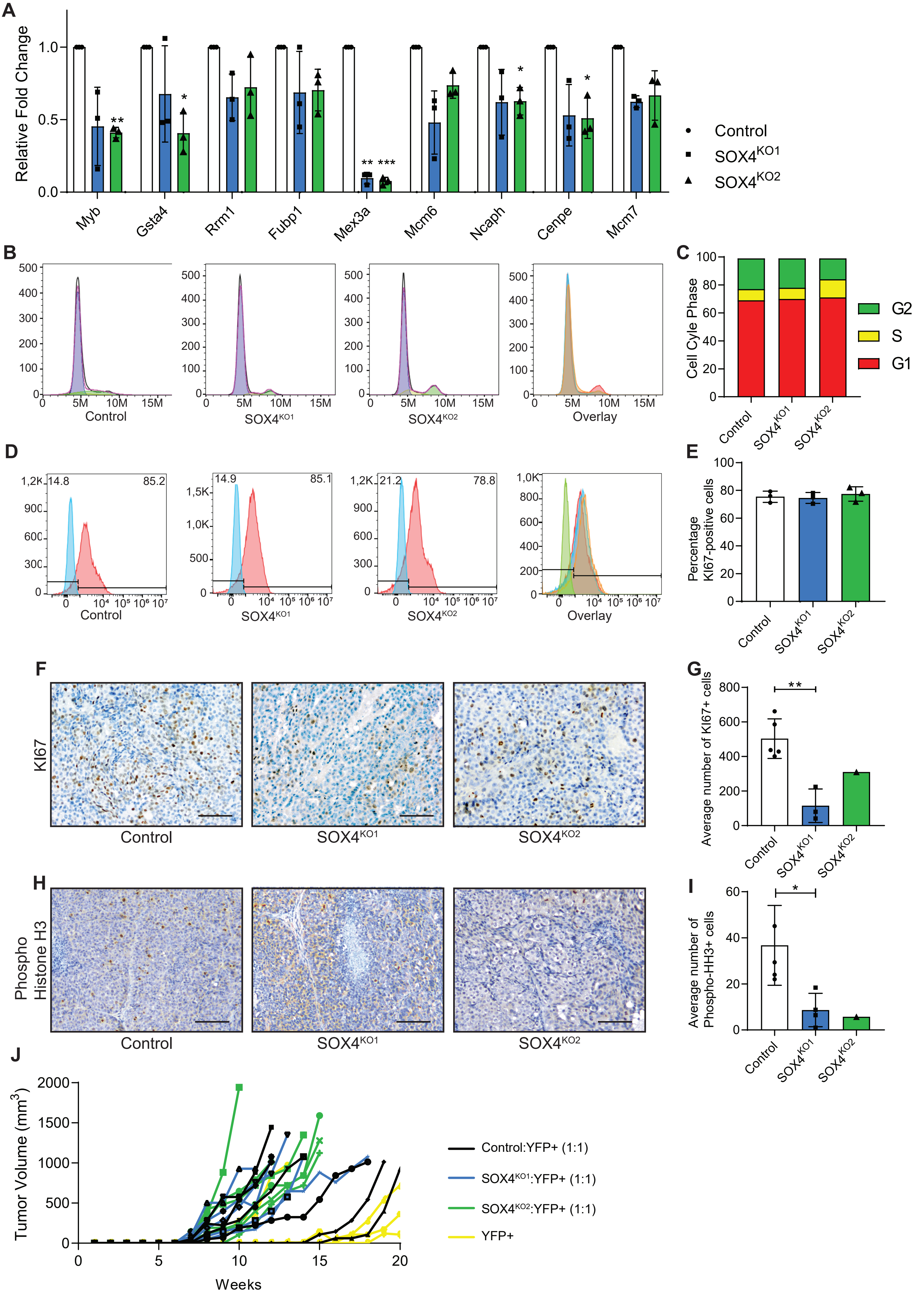

Supplement: Supplementary file 1 — Supplementary Information and Figures [file 41388_2021_2004_MOESM1_ESM.pdf]
